# iCardio: Aplicação de Business Intelligence na Avaliação da Disparidade Regional da Assistência Cardiovascular com Dados do Mundo Real

**DOI:** 10.36660/abc.20250765

**Published:** 2026-05-26

**Authors:** Letícia Cunha Pereira de Souza, Miriam Allein Zago Marcolino, Pedro Henrique Engster, Yohan Casiraghi, Thiago Brusca da Costa Linn, Jerry Eduardo de Almeida de Bairos, Luciana Rodrigues de Lara, Nayê Balzan Schneider, Ana Paula Beck da Silva Etges, Carisi Anne Polanczyk

**Affiliations:** 1 Hospital de Clínicas de Porto Alegre Porto Alegre RS Brasil Hospital de Clínicas de Porto Alegre (HCPA), Porto Alegre, RS – Brasil; 2 Departamento de Bioquímica Instituto de Ciências Básicas da Saúde Universidade Federal do Rio Grande do Sul Porto Alegre RS Brasil Programa de Pós-graduação em Ciências Biológicas: Bioquímica, Departamento de Bioquímica, Instituto de Ciências Básicas da Saúde, Universidade Federal do Rio Grande do Sul, Porto Alegre, RS – Brasil; 3 Instituto Nacional de Avaliação de Tecnologia em Saúde Porto Alegre RS Brasil Instituto Nacional de Avaliação de Tecnologia em Saúde INCT/IATS, Porto Alegre, RS – Brasil; 4 Faculdade de Medicina Universidade Federal de Ciências da Saúde de Porto Alegre Porto Alegre RS Brasil Faculdade de Medicina, Universidade Federal de Ciências da Saúde de Porto Alegre, Porto Alegre, RS – Brasil; 5 Faculdade de Medicina Universidade Federal do Rio Grande do Sul Porto Alegre RS Brasil Faculdade de Medicina, Universidade Federal do Rio Grande do Sul, Porto Alegre, RS – Brasil; 6 Programa de Pós-Graduação em Epidemiologia Universidade Federal do Rio Grande do Sul Porto Alegre RS Brasil Programa de Pós-Graduação em Epidemiologia, Universidade Federal do Rio Grande do Sul, Porto Alegre, RS – Brasil; 7 Programa de Pós-Graduação em Cardiologia e Ciências Cardiovasculares Universidade Federal do Rio Grande do Sul Porto Alegre RS Brasil Programa de Pós-Graduação em Cardiologia e Ciências Cardiovasculares, Universidade Federal do Rio Grande do Sul, Porto Alegre, RS – Brasil

**Keywords:** Cardiologia, Indicadores de Qualidade em Assistência à Saúde, Dados de Saúde Coletados Rotineiramente

## Abstract

**Fundamento:**

A avaliação da assistência cardiovascular no Brasil é desafiada pela extensão territorial e pelo grande volume de dados. Ferramentas de *Business Intelligence* (BI) podem facilitar a visualização e o uso dessas informações na tomada de decisões em saúde pública.

**Objetivos:**

Demonstrar a aplicação prática do painel interativo *iCardio* na análise de desigualdades regionais da assistência cardiovascular de alta complexidade no Brasil.

**Métodos:**

Estudo descritivo, baseado em dados secundários disponibilizados pelo painel *iCardio*, que integra informações assistenciais do Sistema Único de Saúde (SUS). Analisaram-se variáveis de 2019, por localidade. Consideraram-se o volume de atendimentos, as taxas de mortalidade, as reinternações e o deslocamento.

**Resultados:**

As análises dos procedimentos de cirurgias cardiovasculares e cardiologia intervencionista indicaram que o Sudeste apresentou as menores taxas de mortalidade (hospitalar: 5,65%, até 30 dias após o procedimento: 5,73% e até 30 dias após a alta: 6,77%). O Norte concentrou as taxas de mortalidade mais altas (hospitalar: 7,56%, até 30 dias após o procedimento: 7,7% e até 30 dias após a alta: 8,7%). Sul e Centro-Oeste registraram as maiores taxas de reinternação em até 30 dias do procedimento (9,79% e 10,3% respectivamente) e em até 30 dias da alta (11,7% e 11,97%). O Sul se destacou ainda por apresentar a maior taxa padronizada de procedimentos (10,54), quase o dobro do Sudeste que teve a segunda maior taxa (6,02). O Nordeste destacou-se pelo maior deslocamento médio dos pacientes (94,59 km).

**Conclusões:**

O uso de BI por meio do painel *iCardio* permite uma análise ágil e acessível de dados assistenciais, com potencial de apoiar decisões baseadas em evidências, otimização de recursos e identificação de desigualdades regionais na assistência cardiovascular.

## Graphical abstract

**Figura Central:**
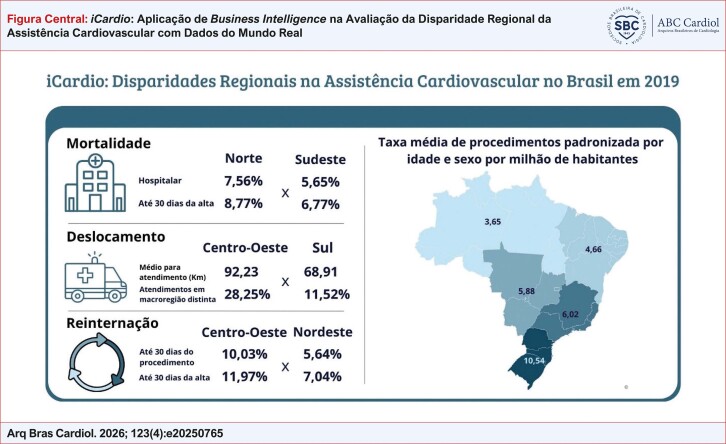
: iCardio: Aplicação de Business Intelligence na Avaliação da Disparidade Regional da Assistência Cardiovascular com Dados do Mundo Real Foram consideradas as regiões com maiores e menores valores para aspecto.

## Introdução

Atualmente, as doenças cardiovasculares (DCV) representam uma carga significativa para os sistemas de saúde em todo o mundo. No Brasil, as DCV se destacam tanto em termos de mortalidade quanto de custos financeiros, com mais de R$1 bilhão sendo gastos anualmente em procedimentos cardiovasculares pelo Sistema Único de Saúde (SUS).^1^ Além disso, a literatura em cardiologia aponta desigualdades regionais na oferta de serviços de alta complexidade,^2^ no volume de atendimentos em centros especializados,^3^ na cobertura da saúde suplementar^4^ e na distribuição de profissionais de saúde no território brasileiro.^5^ Dada a magnitude do impacto desses fatores, a busca por maior eficiência nos investimentos em saúde é de interesse público. Nesse contexto, o uso de painel de indicadores emerge como uma ferramenta promissora, simplificando o acesso e a compreensão das informações e potencialmente melhorando a gestão de recursos e a tomada de decisões.^6-8^

Apesar de seu potencial, há uma escassez de estudos sobre a aplicação de painéis com dados específicos de DCV no Brasil, o que limita a exploração sistemática de informações já disponíveis para subsidiar decisões em saúde pública. Nesse contexto, este estudo se propõe a contribuir com esse campo ao analisar dados do mundo real disponíveis no painel interativo iCardio,^9^ que representa uma iniciativa de organização e visualização de dados de cardiologia intervencionista e cirurgia cardiovascular referentes aos anos de 2019 e 2020. O painel foi desenvolvido com base em dados públicos do Sistema Único de Saúde (SUS), especificamente do Sistema de Informações Hospitalares (SIH) e do Sistema de Informação sobre Mortalidade (SIM).

O *iCardio* fornece parâmetros epidemiológicos detalhados, incluindo o perfil dos pacientes, locais de atendimento, tempo de internação, mortalidade e recursos investidos nos procedimentos. Assim, o objetivo deste estudo foi explorar a plataforma *iCardio* como ferramenta para avaliar a variabilidade dos serviços cardiovasculares entre as regiões do Brasil.

## Métodos

Este é um estudo descritivo ecológico, que teve como fonte de dados o painel de indicadores *iCardio,* plataforma online desenvolvida por pesquisadores do Instituto de Avaliação de Tecnologia em Saúde (IATS) sediado no Hospital de Clínicas de Porto Alegre (https://ror.org/010we4y38), como parte de projeto aprovado em edital do Conselho Nacional de Desenvolvimento Científico e Tecnológico (CNPq – 407282/2022-0) a partir de bases de dados administrativas do Sistema Único de Saúde (SUS). A concepção, arquitetura, fluxos de processamento e validação dos dados do painel *iCardio* estão descritos em publicação prévia.^10^

O painel consolida 17 indicadores quantitativos principais relativos a aspectos clínicos, de produção assistencial, de equidade e sustentabilidade financeira, ao nível hospitalar, estadual e nacional. Esses indicadores estão relacionados a procedimentos de cirurgias cardiovasculares e cardiologia intervencionista realizadas no SUS, nos anos de 2019 e 2020, registrados nos sistemas de dados SIH e SIM. Um total de 165 foram incluídos (Arquivo Suplementar 1) com base nos códigos que começam com os formulários organizacionais 04.06.01 (cirurgia cardiovascular) e 04.06.03 (cardiologia intervencionista) da Tabela de Procedimentos, Medicamentos, Órteses, Próteses e Materiais Especiais do SUS (SIGTAP). Incluíram-se dados de todos os centros que executaram um ou mais procedimento listados acima, o que os classifica como centros de Alta Complexidade Cardiovascular (ACC), que corresponde a unidades e centros de referência qualificados para realizar procedimentos classificados como de alta complexidade, alta tecnologia e alto custo, de acordo com as normas vigentes do SUS.^11^

A base de dados do *iCardio* permite estratificação de dados por sexo, faixa etária, raça/cor, região de residência, tipo de atendimento (urgência/eletivo), diagnóstico (CID-10) e procedimento realizado. Todos os dados são apresentados de forma agregada, sem possibilidade de identificação individual. Para este estudo, foram selecionados os indicadores apresentados na Tabela 1, analisados para cada uma das regiões brasileiras, considerando a análise por localidade disponível no painel. A taxa média padronizada dos procedimentos realizados por idade e sexo expressa por milhão de habitantes foi utilizada para a comparação entre regiões que apresentam populações com diferentes pirâmides etárias e distribuição de sexo. Conforme o material descritivo do desenvolvimento do painel^10,12^ o método de padronização direta foi aplicado, estimando as taxas de procedimento esperadas por região e estado de residência dos pacientes, sexo e faixa etária as quais foram projetadas em função da população de referência definida pelo número de habitantes nessas classes estimado para 2020 pelo Instituto Brasileiro de Geografia e Estatística.

**Tabela 1:** – Indicadores de atendimento, desfechos clínicos e custos das internações hospitalares selecionados por região do Brasil, 2019

Indicador	Medida/Unidade	Finalidade da análise
Quantidade de pacientes (intervenção e cirurgia)	Número absoluto	Dimensionar a população atendida
Quantidade de pacientes intervenção	Número absoluto	Caracterizar volume específico de procedimentos intervencionistas
Quantidade de pacientes cirurgia	Número absoluto	Caracterizar volume específico de procedimentos cirúrgicos
Taxa padronizada por idade e sexo por milhão de habitantes	Taxa por milhão de habitantes	Comparar regiões independentemente da estrutura etária e distribuição de sexo
Homens	Número absoluto	Descrever perfil demográfico dos pacientes
Mulheres	Número absoluto	Descrever perfil demográfico dos pacientes
Principal faixa etária	Faixa etária (%)	Identificar grupo etário mais acometido
Internação em UTI	Percentual (%)	Avaliar gravidade e complexidade do atendimento
Estabelecimentos	Número absoluto	Dimensionar a rede de atendimento disponível
Habitantes/Estabelecimento	Razão (habitantes/unidade)	Estimar acesso e distribuição dos serviços
Deslocamento médio para atendimento	Quilômetros (km)	Avaliar acessibilidade geográfica
Quantidade de pacientes – Região distinta	Número absoluto	Quantificar deslocamentos inter-regionais, considerando a divisão regional do SUS
Pacientes atendidos em uma região distinta	Percentual (%)	Proporção de pacientes que precisaram atendimento fora da região de residência, considerando a divisão regional do SUS
Quantidade de pacientes – Macrorregião distinta	Número absoluto	Quantificar deslocamentos entre macrorregionais, considerando a divisão regional do SUS
Pacientes atendidos em uma macrorregião distinta	Percentual (%)	Proporção de pacientes que precisaram atendimento fora da macrorregião de residência, considerando a divisão regional do SUS
Reinternação em até 30 dias do procedimento	Percentual (%)	Avaliar desfecho precoce após intervenção
Reinternação em até 30 dias da alta	Percentual (%)	Avaliar qualidade do cuidado e acompanhamento pós-alta
Mortalidade hospitalar	Percentual (%)	Mensurar letalidade durante a internação
Mortalidade em até 30 dias do procedimento	Percentual (%)	Avaliar risco de óbito precoce após procedimento
Mortalidade em até 30 dias da alta	Percentual (%)	Avaliar risco de óbito precoce após alta hospitalar
Custo médio por internação	Reais (R$)	Estimar custo médio por paciente atendido
Valor total	Reais (R$)	Dimensionar impacto financeiro agregado

UTI: unidade de terapia intensiva. Fonte: iCardio.

Adicionalmente, se analisou a distribuição das taxas de mortalidade, segundo sexo e faixa etária, com estratificação entre pacientes pediátricos e adultos. E também os desfechos assistências foram analisados de forma individualizada para cada procedimento. Foram analisados dados apenas das internações do ano de 2019.

Os dados foram extraídos do painel *iCardio* e organizados em planilhas eletrônicas para análise descritiva utilizando o Microsoft Excel (Microsoft Corporation, Redmond, WA, EUA). Foram calculadas medidas de frequência (valores absolutos e percentuais) para sumarizar os indicadores selecionados. A comparação entre as diferentes regiões geográficas brasileiras e os perfis de pacientes foi realizada por meio de análise descritiva comparativa. Como os dados utilizados se referem ao conjunto populacional total de internações registradas no SUS para os procedimentos analisados, não foram aplicados testes de hipóteses ou métodos estatísticos inferenciais.

O estudo foi aprovado pelo Comitê de Ética em Pesquisa Institucional do Hospital de Clínicas de Porto Alegre, sob o número do projeto CAAE 6784523.0.0000.5327 e todo o processamento dos dados seguiu a legislação nacional de proteção de dados. Este estudo utilizou apenas dados secundários e anonimizados, disponíveis em domínio público, não envolvendo qualquer intervenção em seres humanos.

## Resultados

### Distribuição territorial da assistência cardiovascular no Brasil

Para cada uma das cinco regiões brasileiras, os dados dos indicadores clínicos estão resumidos na Tabela 2. Ao comparar o número total de pacientes atendidos com o número de atendimentos registrados nas áreas de cardiologia intervencionista e cirurgia cardiovascular, se observa que a soma dos atendimentos nas duas áreas excede o número total de pacientes atendidos; isso ocorre, pois, alguns pacientes foram submetidos a procedimentos em ambas as especialidades.

**Tabela 2:** – Perfil clínico e demográfico das internações por procedimentos cardiovasculares no SUS, Brasil, 2019

Indicadores (2019)	Norte	Nordeste	Centro-Oeste	Sudeste	Sul	Brasil
**Dados clínicos**						
Quantidade de pacientes (intervenção e cirurgia)	4.742	30.693	10.956	71.350	43.188	160.897
Quantidade de pacientes intervenção	2.165	15.128	5.351	40.203	24.665	87.497
Quantidade de pacientes cirurgia	2.653	16.119	5.825	32.647	19.893	77.128
Homens	3.071	17.583	6609	43.931	27.167	98.340
Mulheres	1.669	13.088	4.3364	27.389	16.009	62.480
Taxa padronizada por idade e sexo por milhão de habitantes	3,65	4,66	5,88	6,02	10,54	5,43
**Distribuição etária da quantidade de pacientes**						
<1 ano	88	451	139	921	462	2.061
01 a 08 anos	181	903	306	1.741	780	3.910
09 a 17 anos	82	336	85	576	246	1.324
18 a 26 anos	73	370	92	529	239	1.302
27 a 35 anos	114	635	160	844	423	2.175
36 a 44 anos	209	1.506	448	2.608	1.460	6.229
45 a 53 anos	543	3.696	1.320	8.166	4.801	18.520
54 a 62 anos	1.072	6.796	2.564	17.466	10,395	38.284
63 a 71 anos	1.208	7.737	2.870	19.904	12,397	44.109
72 a 80 anos	813	5.485	2.037	12.875	8.398	29.606
**Distribuição racial dos pacientes**						
Amarela	105	344	97	628	400	1.574
Branca	408	2.484	1.559	38.412	36.553	79.406
Indígena	7	6	9	5	16	43
Parda	3.048	14.770	5.140	21.385	2.724	47.057
Preta	116	595	118	3.527	1.031	5.387
Conflito	39	65	43	436	135	707
Sem informação	1.019	12.429	3.990	6.957	2.329	26.723

Fonte: iCardio. Nota: A soma dos pacientes por faixa etária não corresponde ao total de pacientes atendidos reportado, discrepância explicada pelo processamento específico adotado para ajuste de inconsistências nas datas de nascimento registradas nas Autorizações de Internação Hospitalar (AIHs), garantindo maior confiabilidade na classificação etária.^12^

Considerando todos os atendimentos realizados no país, se observou predominância de atendimento de pacientes brancos do sexo masculino, com idade entre 63 e 71 anos. Entretanto, a predominância de pacientes brancos na análise agregada reflete, em grande medida, a maior concentração de atendimentos nas regiões Sudeste e Sul, onde há, conforme evidenciado no painel, tanto maior volume de procedimentos quanto maior proporção de população autodeclarada branca. Em contraste, nas regiões Norte, Nordeste e Centro-Oeste, se observa predominância de atendimentos a pacientes que se identificam como pardos, evidenciando diferenças regionais no perfil racial dos pacientes atendidos, ainda que não tenham sido observadas diferenças expressivas quanto ao sexo e à distribuição etária entre as regiões.

Na análise estratificada por idade e sexo (Tabela Suplementar 2), a mortalidade hospitalar foi consistentemente mais elevada na população pediátrica em comparação aos adultos, com destaque para a região Norte, onde, entre os homens, a taxa nos pacientes pediátricos é mais que o dobro da observada nos adultos. Entre as mulheres, essa diferença etária é menos pronunciada. Em contraste, ao considerar apenas a população adulta, se observa um padrão inverso: em todas as regiões do país, as mulheres apresentam taxas de mortalidade superiores às dos homens.

Observa-se que a região Sudeste concentra o maior número bruto de atendimentos e procedimentos realizados, seguida pela região Sul. No entanto, ao considerar a taxa padronizada por idade e sexo, se verifica que a região Sul apresenta o maior valor. Embora Sudeste e Sul se destaquem em volume de atendimentos, a maior proporção de internações em UTI (unidade de terapia intensiva) é observada na região Nordeste, conforme apresentado na Tabela 3, que reúne os indicadores assistenciais e econômicos. No total, o país possui 558 estabelecimentos de ACC, com a razão entre o número de habitantes e estabelecimentos variando entre 130.149 e 229.644, a depender da região. As distâncias médias percorridas pelos pacientes para receber atendimento também variam conforme a localidade, com a região Sudeste apresentando um deslocamento médio significativamente menor que o das outras regiões.

**Tabela 3:** – Distribuição regional dos indicadores assistenciais e econômicos das internações hospitalares por procedimentos cardiovasculares, Brasil, 2019

Indicadores (2019)	Norte	Nordeste	Centro-Oeste	Sudeste	Sul	Brasil
**Indicadores assistenciais**						
Internação em UTI (%)	45,79	58,21	49,49	56,04	53,39	54,69
Estabelecimentos	35	93	41	276	113	588
Habitantes/Estabelecimento	229.644	194.685	201.276	204.558	130.149	189.176
Deslocamento médio para atendimento (Km)	90,77	94,59	92,23	45,86	68,91	65,77
Quantidade de pacientes – Região distinta	1.726	14.824	3.544	24.249	14.990	59.327
Pacientes atendidos em uma região distinta (%)	36,38	48,23	32,18	33,88	34,60	36,76
Quantidade de pacientes –Macrorregião distinta	969	7.158	3.112	10.360	4.984	26.579
Pacientes atendidos em uma macrorregião distinta (%)	20,43	23,29	28,25	14,49	11,52	16,49
Reinternação em até 30 dias do procedimento (%)	6,05	5,64	10,30	7,08	9,79	7,75
Reinternação em até 30 dias da alta (%)	7,56	7,04	11,97	8,71	11,70	9,41
Mortalidade hospitalar (%)	7,56	6,28	6,34	5,65	6,58	6,12
Mortalidade em até 30 dias do procedimento (%)	7,70	6,49	6,91	5,73	6,88	6,32
Mortalidade em até 30 dias da alta (%)	8,70	7,52	7,72	6,77	7,81	7,32
**Indicadores econômicos**						
Custo médio por internação (R$)	11.153,55	11.518,30	11.059,58	10.765,24	11.411,49	11.114,04
Valor total (R$)	54.73 M	369.54 M	133.77 M	818.89 M	538.15 M	1.92 B

UTI: unidade de terapia intensiva. Fonte: iCardio.

Com base na divisão em regiões e macrorregiões de saúde, se observa que os percentuais atendidos fora da região e da macrorregião variam entre os tipos de atendimentos. Entre os pacientes submetidos à cirurgia cardiovascular, 41,44% foram atendidos fora da região de residência e 21,16% fora da macrorregião. Já na cardiologia intervencionista, esses valores foram menores, com 32,46% dos atendimentos ocorrendo fora da região e 12,23% fora da macrorregião.

As regiões Centro-Oeste e Sul apresentam elevadas taxas de reinternação, tanto no período de até 30 dias após o procedimento quanto no intervalo de até 30 dias após a alta. Em relação à mortalidade, se observam os maiores valores brutos na região Norte nos três desfechos analisados - mortalidade hospitalar, em até 30 dias após o procedimento e em até 30 dias após a alta - além do maior tempo médio de internação. Já a região Sudeste apresenta os menores percentuais brutos para os três tipos de mortalidade.

Em uma análise por grupo de procedimentos (Arquivo Suplementar 2), os procedimentos com maiores taxas de mortalidade hospitalar no Brasil foram cateterismo (31,87%), correção de anomalias cardíacas (21,55%) e cardiorressecção (17,01%). Entre regiões, a septectomia ventricular alcança 57,14% no Centro-Oeste, correção de anomalias cardíacas atinge 31,75% no Norte, e anastomose registra 32% no Nordeste. Cardiorressecção apresenta 22,73% no Norte e plástica cardíaca 18,76% no Sul. O Nordeste se destaca em mortalidade 30 dias da alta para anastomose (48%). Entretanto, é importante salientar que a análise considerou a ocorrência de óbito durante internações associadas aos procedimentos, e não a causa básica do óbito, de modo que os resultados refletem mortalidade hospitalar associada à internação e não mortalidade atribuível ao procedimento.

Quanto aos recursos financeiros, a taxa de reembolso para os procedimentos realizados foi de cerca de R$1,9 bilhão no período analisado. Embora o custo médio por internação apresente variações modestas entre as regiões, os gastos regionais variam amplamente, com valores que vão de R$54,73 milhões a R$818,89 milhões, acompanhando o volume de atendimentos prestados nas regiões.

## Discussão

Com base nos dados do painel *iCardio* para o ano de 2019, foi realizada uma análise das variações regionais nos atendimentos cardiovasculares, visando aprofundar a compreensão das desigualdades na assistência cardiovascular no Brasil, estando os resultados resumidos na Figura Central, visto que o país apresenta grande extensão territorial, com alta heterogeneidade entre suas regiões em termos de aspectos naturais, econômicos e sociais.^13^ O SUS vem buscando reduzir as desigualdades na saúde entre as regiões e melhorar a cobertura e o acesso aos cuidados de saúde em todo o país, embora persistam grandes variações em termos de infraestrutura, recursos humanos, capacidade de gestão e acesso a serviços de saúde eficazes.^14^ Assim, é fundamental considerar como essas particularidades influenciam os indicadores de saúde.

Considerando o perfil clínico geral do país, estudos comparativos entre pacientes pediátricos e adultos reforçam os dados apresentados, a literatura indica que a taxa de mortalidade em procedimentos e cirurgias cardiovasculares de alta complexidade é significativamente maior em crianças,^15^ quando comparada a adultos submetidos a procedimentos similares. Além disso, a análise de bancos de dados internacionais indica que a mortalidade pediátrica está fortemente associada à complexidade do procedimento, idade e peso ao nascer,^16^ enquanto em adultos o risco é mais influenciado por comorbidades adquiridas e idade avançada.^17^ Ao passo que a diferença entre sexos é principalmente explicada diferentes perfis de risco pré-operatório,^18^ estando a mortalidade em mulheres mais associada a doenças sistemáticas e nos homens a dano cardíaco direto. Além disso, há relato de que alguns dos sintomas indicativos de DCV tendem a ser diferentes entre homens e mulheres, sendo um fator contribuinte para atrasos no diagnóstico, menor acesso a procedimentos invasivos e pior prognóstico em cirurgias cardiovasculares de alta complexidade.^19-21^Porém, maiores investigações seriam necessárias para identificar mais precisamente as causas das diferenças observadas no presente estudo.

No geral, no estudo, se observou que a região Norte apresenta indicadores de desfechos duros inferiores aos observados em outras regiões. Uma análise prévia de indicadores na área de cardiologia indicou uma tendência da região Norte de apresentar piores rendimentos, o que pode ser explicado por fatores socioeconômicos.^1^ Essa região apresenta ainda algumas limitações com relação à disponibilidade de dados precisos nos sistemas de saúde.^22^ Assim como o Norte, o Nordeste tende apresentar dados preocupantes com relação ao acesso a cuidados médicos e a sua qualidade.^1,22^ Na análise do painel, se observou que essa região apresentou a maior porcentagem de internações em UTI e maior deslocamento médio. Ao passo que, a região Sudeste se destaca por concentrar o maior número de centros de ACC e minimizar o deslocamento dos pacientes, apresentando os melhores indicadores de saúde e o menor custo por internação. Isso pode ser explicado pelo fato de que hospitais maiores ou com alto fluxo diluem custos fixos em mais casos, aproveitam melhor a capacidade instalada e ganham eficiência no uso de recursos.^23^

Sul e Centro-Oeste se destacam por apresentarem as maiores taxas de reinternação, fatores que influenciam esse desfecho incluem qualidade da alta hospitalar, continuidade do cuidado ambulatorial e adesão ao tratamento. Além disso, o perfil dos pacientes é um fator crítico no que se refere a internações, comorbidades, idade avançada e maior prevalência de tabagismo na população também contribuem para esse cenário, aumentando custos e pressão sobre leitos hospitalares.^24^ Estudos prévios apontam que a região Sul do Brasil apresenta o maior percentual de fumantes em comparação com outras regiões^25^ e a segunda população mais idosa do país, com 12,1% de seus moradores com 65 anos ou mais.^26^Mas essas regiões divergem no que diz respeito ao número de pacientes atendidos em uma macrorregião distinta. O centro-Oeste apresenta a maior porcentagem de pacientes atendidos em uma macrorregião distinta, ao passo que o Sul apresenta a menor porcentagem. Essa divisão territorial em regiões e macrorregiões de saúde proposta pelo SUS visa otimizar o planejamento e a integração das ações e serviços de saúde, assegurando que os recursos e necessidades da população sejam considerados de forma regionalizada.^27,28^

Além disso, a análise nacional dos dados do painel *iCardio* evidenciou variações no deslocamento de pacientes entre as regiões e macrorregiões conforme o tipo de procedimento. Observou-se que os procedimentos de cardiologia intervencionista, em geral, são realizados dentro da própria região ou macrorregião de residência, sugerindo uma oferta mais distribuída desse tipo de serviço. Em contrapartida, as cirurgias cardiovasculares demandam, com maior frequência, o deslocamento dos pacientes para outras regiões ou macrorregiões, indicando que a macrorregião de origem muitas vezes não possui capacidade instalada suficiente para atender a demandas de maior complexidade.

A análise dos indicadores econômicos evidenciou diferenças expressivas no gasto total entre as regiões, as quais se relacionam primordialmente ao volume de internações realizadas, dado que não se observaram variações relevantes no custo médio por internação entre elas. Esse padrão é consistente com a lógica de financiamento do SUS, em que a maior parte da produção hospitalar é remunerada por procedimentos com valores previamente definidos em tabela, o que tende a equalizar o custo médio por internação regionalmente, ainda que existam diferenças no perfil de casos e na complexidade assistencial. Dessa forma, o gasto total passa a refletir, sobretudo, o tamanho populacional^29^ e a carga de doença.^30^ Além disso, outro fator que influencia é a capacidade instalada, incluindo a oferta de leitos e serviços de alta complexidade que atuam como polos de referência e concentram internações oriundas de outras regiões.^5^ Sendo que o alto deslocamento médio para atendimento da região Nordeste pode justificar por que, apesar de essa ser a segunda região mais populosa, não é a segunda com maior gasto total.

Os dados de mundo real analisados foram disponibilizados por uma plataforma de *Business Intelligence* (BI) que visa facilitar a análise e a tomada de decisões em saúde pública com informações mais acessíveis e precisas. O uso desses dados como evidências ganhou destaque desde 2007, com o relatório da *International Society for Pharmacoeconomics and Outcomes Research* (ISPOR) incentivando o uso de dados e evidências do mundo real na avaliação de tecnologias em saúde e no apoio à tomada de decisões clínicas e políticas,^31^ e hoje é incentivado por agências regulatórias como FDA (*Food and Drug Administration*) e ANVISA (Agência Nacional de Vigilância Sanitária).^32,33^ No Brasil, se conta com o Departamento de Informática do Sistema Único de Saúde (DATASUS) que gerencia os sistemas nacionais SIH e SIM, utilizados neste estudo. Além do SIGTAP que serve de apoio gerencial logístico de qualificação das informações.^34^

Diversos estudos já demonstraram o potencial de ferramentas de BI aplicadas à saúde, com exemplos nas áreas de pneumologia, cardiologia e neurologia, onde painéis automatizados foram utilizados para análise da efetividade de terapias, taxas de mortalidade e impacto econômico.^35-38^ Exemplos relevantes incluem o painel interativo da Universidade Johns Hopkins^39^ e o painel do Ministério da Saúde para monitorar casos e óbitos de COVID-19.^40^ Esses exemplos reforçam o valor das plataformas baseadas em BI, tanto para a resposta a emergências sanitárias quanto para a gestão contínua dos serviços de saúde, evidenciando a importância do uso sistemático de dados do mundo real para gerar evidências que qualifiquem o planejamento e a tomada de decisões em saúde.

Os sistemas de informação do SUS foram criados com propósito administrativo e se caracterizam pela fragmentação das informações. O DATASUS e outras iniciativas de transparência disponibilizam acesso público ao conteúdo de cada sistema separadamente, não havendo identificador que possibilite a conexão de eventos de saúde e mortalidade geral ao nível do paciente. Nesse aspecto, o *iCardio*, utilizando os dados pareados entre sistemas^41^ consegue disponibilizar, em um único ambiente, indicadores clínico-epidemiológicos, assistenciais, geográficos e econômicos, já calculados e estratificáveis. Assim, o uso do *iCardio* apresenta potencial relevante para subsidiar a tomada de decisão clínicas e em políticas públicas. Embora neste estudo a análise tenha sido conduzida em nível regional, o painel permite também outras avaliações, possibilitando o monitoramento de desempenho de serviços, a identificação de variabilidade injustificada e o apoio a iniciativas de melhoria da qualidade. Dessa forma, a ferramenta pode orientar a organização de redes de atenção, a definição de centros de referência e a alocação de recursos, contribuindo para decisões mais informadas, eficientes e equânimes no SUS.

Embora o painel *iCardio* disponibilize dados de 2019 e 2020, se analisou apenas 2019, já que a pandemia de COVID-19 alterou profundamente o perfil de internações e o uso de recursos, podendo distorcer a análise e comprometer a comparabilidade regional. Ressalta-se que este estudo apresenta algumas limitações que devem ser consideradas na interpretação dos resultados. Primeiramente, a plataforma *iCardio* ainda não integra ferramentas oficiais e está disponível apenas em português. Além disso, a análise se baseia em dados secundários do SUS (SIH e SIM), sujeitos a subnotificação e inconsistências; e, na análise principal, os dados foram avaliados de forma agregada, sem estratificação por tipo específico de procedimento (por exemplo, angioplastia, cirurgia de revascularização do miocárdio ou cirurgias valvares), e os desfechos de reinternação e mortalidade não foram ajustados para a complexidade dos procedimentos. No entanto, análises adicionais estratificadas por tipo de procedimento, sexo e faixa etária foram realizadas e estão disponíveis no material suplementar. Por fim, o uso exclusivo de dados de 2019, embora evite distorções da pandemia, restringe as conclusões a um período pré-COVID-19 e configura uma limitação temporal relevante, uma vez que a plataforma não tem perspectivas de atualização, impossibilitando a análise de tendências mais recentes.

## Conclusões

Este artigo demonstra uma aplicação prática da automatização na avaliação da prestação de cuidados cardiovasculares com base em dados do mundo real, ampliando a maturidade digital para uso dessa informação. O uso de painéis interativos, como o *iCardio*, permite tornar a análise de grandes volumes de dados mais acessível, rápida e intuitiva, o que potencializa seu uso por gestores, pesquisadores e formuladores de políticas públicas. Essa abordagem tem o potencial de transformar a gestão em saúde, ao apoiar decisões mais assertivas, baseadas em evidências, e contribuir para a identificação de desigualdades regionais, otimização de recursos e melhoria da qualidade da assistência cardiovascular no país. Esses achados reforçam a existência de disparidades na oferta e utilização de serviços de alta complexidade, apontadas previamente na literatura, e destacam a importância de estratégias que promovam maior equidade no acesso e na qualidade do cuidado cardiovascular no país.

## Material suplementar

Material Suplementar 1

Material Suplementar 2

Material Suplementar 3

Tabela Suplementar
