# Supplementary material for: iCardio: Aplicação de Business Intelligence na Avaliação da Disparidade Regional da Assistência Cardiovascular com Dados do Mundo Real
Source: Arq Bras Cardiol. 2026 May 26;123(4):e20250765. [Article in Portuguese] doi: 10.36660/abc.20250765 (PMC13398835; doi:10.36660/abc.20250765)
Supplement: Material Suplementar 1 [file 0066-782x-abc-123-4-e20250765-suppl01.pdf]

Código do procedimento	Nome do procedimento	Forma de organização	Descrição	Valor Serviço Hospitalar	Valor Serviço Profissional	Total Hospitalar	Tempo médio de permanência (dias)	Admite permanência à maior	Inclui valor da anestesia	Grupo de procedimentos
04.06.01.001-3	ABERTURA DE COMUNICAÇÃO INTER-ATRIAL	01 - Cirurgia cardiovascular	PROCEDIMENTO QUE CONSISTE EM CRIAR OU AMPLIAR A COMUNICAÇÃO ENTRE OS ÁTRIOS DIREITO E ESQUERDO, ATRAVÉS DE RESSECÇÃO DO SEPTO INTERATRIAL, PARA PROMOVER MISTURA DO SANGUE, EM CRIANÇAS COM CARDIOPATIA CONGÊNITA COMPLEXA, ATRAVÉS DE CIRURGIA (CIRURGIA DE BLALOCK-HANLON)	R$ 7.960,32	R$ 4.286,33	R$ 12.246,65	5	Sim	Sim	CARDIORRESSECÇÃO
04.06.01.002-1	ABERTURA DE ESTENOSE AORTICA VALVAR	01 - Cirurgia cardiovascular	PROCEDIMENTO QUE CONSISTE EM AUMENTAR O FLUXO DE SANGUE DO VENTRÍCULO ESQUERDO PARA A AORTA, TRATANDO VÁLVULA COM FLUXO RESTRITIVO, SEM PRÓTESE VALVAR.	R$ 4.079,80	R$ 3.365,37	R$ 7.445,17	5	Sim	Sim	ABERTURA DE ESTENOSE
04.06.01.003-0	ABERTURA DE ESTENOSE PULMONAR VALVAR	01 - Cirurgia cardiovascular	PROCEDIMENTO QUE CONSISTE EM AUMENTAR O FLUXO DE SANGUE DO VENTRÍCULO DIREITO PARA A ARTÉRIA PULMONAR, TRATANDO VÁLVULA COM FLUXO RESTRITIVO, SEM PRÓTESE VALVAR.	R$ 4.079,80	R$ 3.365,37	R$ 7.445,17	5	Sim	Sim	ABERTURA DE ESTENOSE
04.06.01.004-8	AMPLIAÇÃO DE VIA DE SAÍDA DO VENTRICULO DIREITO E/OU RAMOS PULMONARES	01 - Cirurgia cardiovascular	PROCEDIMENTO QUE CONSISTE EM AUMENTAR O FLUXO DE SANGUE ENTRE O VENTRÍCULO DIREITO E AS ARTÉRIAS PULMONARES, ATRAVÉS DE AMPLIAÇÃO E ALARGAMENTO DE PARTE DO VENTRÍCULO DIREITO E/OU ANEL VALVAR PULMONAR E/OU ARTÉRIAS PULMONARES, COM USO DE RETALHOS DE ENXERTOS ORGÂNICOS E/OU INORGÂNICOS COM OU SEM PRÓTESES VALVARES.	R$ 7.357,69	R$ 3.829,47	R$ 11.187,16	8	Sim	Sim	SEPTECTOMIA VENTRICULAR
04.06.01.005-6	AMPLIAÇÃO DE VIA DE SAÍDA DO VENTRÍCULO ESQUERDO	01 - Cirurgia cardiovascular	PROCEDIMENTO QUE CONSISTE EM AUMENTAR O FLUXO DE SANGUE ENTRE O VENTRÍCULO ESQUERDO E A AORTA, ATRAVÉS DE AMPLIAÇÃO E ALARGAMENTO DE PARTE DO VENTRÍCULO ESQUERDO E/OU ANEL VALVAR AÓRTICO, COM USO DE RETALHOS DE ENXERTOS ORGÂNICOS E/OU INORGÂNICOS, MAIS ADIÇÃO DE PRÓTESES VALVARES.	R$ 8.528,04	R$ 4.321,19	R$ 12.849,23	8	Sim	Sim	SEPTECTOMIA VENTRICULAR
04.06.01.006-4	ANASTOMOSE CAVO-PULMONAR BIDIRECIONAL	01 - Cirurgia cardiovascular	PROCEDIMENTO QUE CONSISTE EM CRIAR UM ATALHO DO SANGUE ENTRE A VEIA CAVA SUPERIOR E AS ARTÉRIAS PULMONARES, ATRAVÉS DA CONECÇÃO DA VEIA CAVA SUPERIOR DIRETAMENTE NAS ARTÉRIAS PULMONARES, EM CRIANÇAS COM HIPODESENVOLVIMENTO DE UM DOS VENTRÍCULOS. ATUALMENTE PODE SER FEITA PARA DESCOMPRESSÃO DO VENTRÍCULO DIREITO EM OUTRAS DOENÇAS COMO ANOMALIA DE EBSTEIN.	R$ 6.508,73	R$ 1.923,03	R$ 8.431,76	8	Sim	Sim	ANASTOMOSE
04.06.01.007-2	ANASTOMOSE CAVO-PULMONAR TOTAL	01 - Cirurgia cardiovascular	PROCEDIMENTO QUE CONSISTE EM CRIAR UM ATALHO DO SANGUE ENTRE AS 2 VEIAS CAVAS (SUPERIOR E INFERIOR) E AS ARTÉRIAS PULMONARES, ATRAVÉS DA CONECÇÃO DIRETAMENTE NAS ARTÉRIAS PULMONARES, EM CRIANÇAS COM HIPODESENVOLVIMENTO DE UM DOS VENTRÍCULOS. A CONECÇÃO DA VEIA CAVA INFERIOR NAS ARTÉRIAS PULMONARES NECESSITA ENXERTO TUBULAR ORGÂNICO E/OU INORGÂNICO E/OU PATCH.	R$ 10.762,50	R$ 5.795,19	R$ 16.557,69	8	Sim	Sim	ANASTOMOSE
04.06.01.008-0	ANASTOMOSE SISTEMICO-PULMONAR	01 - Cirurgia cardiovascular	PROCEDIMENTO QUE CONSISTE EM AUMENTAR O FLUXO DE SANGUE PARA OS PULMÕES, CRIANDO UM DESVIO DO SANGUE DA AORTA E SEUS RAMOS PARA AS ARTÉRIAS PULMONARES, ATRAVÉS DE CONEXÃO DIRETA NAS ARTÉRIAS PULMONARES OU COM USO DE ENXERTOS TUBULARES.	R$ 3.631,92	R$ 1.923,03	R$ 5.554,95	5	Sim	Sim	ANASTOMOSE
04.06.01.010-2	CARDIORRAFIA	01 - Cirurgia cardiovascular	SUTURA DE CORAÇÃO.	R$ 1.175,18	R$ 693,36	R$ 1.868,54	5	Sim	Sim	OUTROS PROCEDIMENTOS CIRÚRGICOS CARDIOVASCULARES
04.06.01.011-0	CARDIOTOMIA P/ RETIRADA DE CORPO ESTRANHO	01 - Cirurgia cardiovascular	PROCEDIMENTO QUE CONSISTE EM INCISAR UMA DAS CÂMARAS DO CORAÇÃO PARA RETIRAR CORPO ESTRANHO, EXEMPLO: PROJÉTEIS DE ARMA DE FOGO, DISPOSITIVOS USADOS EM HEMODINÂMICA (STENTS, COILS),ETC.	R$ 1.175,18	R$ 561,87	R$ 1.737,05	5	Sim	Sim	OUTROS PROCEDIMENTOS CIRÚRGICOS CARDIOVASCULARES
04.06.01.012-9	COLOCAÇÃO DE BALÃO INTRA-AÓRTICO	01 - Cirurgia cardiovascular	COLOCAÇÃO DE UM CATETER BALÃO POR PUNÇÃO OU DISSECÇÃO DA ARTÉRIA FEMORAL OU SUBCLÁVIA PARA AUXÍLIO À FUNÇÃO DO VENTRÍCULO ESQUERDO.	R$ 45,00	R$ 0,00	R$ 45,00	-	Não		CATETERISMO
04.06.01.013-7	CORREÇÃO DE ANEURISMA / DISSECÇÃO DA AORTA TORACO-ABDOMINAL	01 - Cirurgia cardiovascular	A CIRURGIA CONSISTE EM CORRIGIR A AORTA ANEURISMÁTICA OU DISSECADA COM A INTERPOSIÇÃO DE UM ENXERTO TUBULAR, ORGÂNICO OU INORGÂNICO, VALVULADO OU NÃO, ANASTOMOSANDO OU NÃO, DEPENDENDO DA DOENÇA, OS VASOS DA BASE (TRONCO BRAQUIO-CEFÁLICO DIREITO, CARÓTIDA E SUBCLÁVIA ESQUERDA) NO TUBO. DEPENDENDO DA DOENÇA (DISSECÇÃO) ALGUMAS VEZES É NECESSÁRIO EXTENDER O NOVO TUBO À AORTA TORÁCICA DESCENDENTE E ATÉ A ABDOMINAL. FEITA COM TORACOTOMIA E COM CIRCULAÇÃO EXTRACORPÓREA.	R$ 2.983,09	R$ 7.132,96	R$ 10.116,05	5	Sim	Sim	CORREÇÃO DE ANOMALIAS CARDÍACAS
04.06.01.015-3	CORREÇÃO DE ATRESIA PULMONAR E COMUNICAÇÃO INTERVENTRICULAR	01 - Cirurgia cardiovascular	PROCEDIMENTO QUE CONSISTE EM FECHAR A COMUNICAÇÃO ENTRE OS DOIS VENTRÍCULOS COM USO DE RETALHO DE ENXERTO ORGÂNICO OU INORGÂNICO, MAIS A CRIAÇÃO DE UMA DERIVAÇÃO (PONTE) ENTRE O VENTRÍCULO DIREITO E A ARTÉRIA PULMONAR USANDO UM ENXERTO TUBULART VALVADO ORGÂNICO OU INORGÂNICO.	R$ 14.474,15	R$ 7.793,77	R$ 22.267,92	8	Sim	Sim	CORREÇÃO DE ANOMALIAS CARDÍACAS
04.06.01.016-1	CORREÇÃO DE ÁTRIO ÚNICO	01 - Cirurgia cardiovascular	PROCEDIMENTO QUE CONSISTE EM CRIAR UMA SEPTAÇÃO ENTRE OS DOIS ÁTRIOS, IMPEDINDO A PASSAGEM DE SANGUE DE UM ÁTRIO PARA O OUTRO.	R$ 9.545,53	R$ 5.139,90	R$ 14.685,43	8	Sim	Sim	CORREÇÃO DE ANOMALIAS CARDÍACAS

04.06.01.017-0	CORREÇÃO DE BANDA ANÔMALA DO VENTRÍCULO DIREITO	01 - Cirurgia cardiovascular	PROCEDIMENTO QUE CONSISTE EM RESSECAR SEGMENTOS DE MÚSCULO DO VENTRÍCULO DIREITO QUE ATRAPALHA A PASSAGEM DE SANGUE PARA AS ARTÉRIAS PULMONARES.	R$ 7.116,60	R$ 3.832,02	R$ 10.948,62	5	Sim	Sim	CARDIORRESSECÇÃO
04.06.01.018-8	CORREÇÃO DE COARCTAÇÃO DA AORTA	01 - Cirurgia cardiovascular	PROCEDIMENTO QUE CONSISTE EM RESSECAR OU AMPLIAR REGIÃO DA AORTA QUE ENCONTRA-SE COM OBSTRUÇÃO PARCIAL, COM OU SEM USO DE ENXERTOS TUBULARES OU RETALHOS.	R$ 3.706,55	R$ 1.923,03	R$ 5.629,58	5	Sim	Sim	CARDIORRESSECÇÃO
04.06.01.019-6	CORREÇÃO DE COMUNICAÇÃO INTERVENTRICULAR	01 - Cirurgia cardiovascular	PROCEDIMENTO QUE CONSISTE EM CORRIGIR A COMUNICAÇÃO ENTRE OS DOIS VENTRÍCULOS, IMPEDINDO A PASSAGEM DE SANGUE DE UM VENTRÍCULO PARA O OUTRO, ATRAVÉS DO USO DE RETALHOS DE ENXERTO ORGÂNICO OU INORGÂNICO.	R$ 10.220,38	R$ 3.365,37	R$ 13.585,75	8	Sim	Sim	CORREÇÃO DE ANOMALIAS CARDÍACAS
04.06.01.020-0	CORREÇÃO DE COMUNICAÇÃO INTER-VENTRICULAR E INSUFICIÊNCIA AORTICA	01 - Cirurgia cardiovascular	PROCEDIMENTO QUE CONSISTE EM CORRIGIR A COMUNICAÇÃO ENTRE OS DOIS VENTRÍCULOS, IMPEDINDO A PASSAGEM DE SANGUE DE UM VENTRÍCULO PARA O OUTRO, ATRAVÉS DO USO DE RETALHOS DE ENXERTO ORGÂNICO OU INORGÂNICO, MAIS A CORREÇÃO DE DEFEITO NA VALVA AÓRTICA, ATRAVÉS DE PLÁSTICA DA MESMA.	R$ 6.508,73	R$ 3.365,37	R$ 9.874,10	8	Sim	Sim	CORREÇÃO DE ANOMALIAS CARDÍACAS
04.06.01.021-8	CORREÇÃO DE COR TRIATRIATUM	01 - Cirurgia cardiovascular	PROCEDIMENTO QUE CONSISTE EM RESSECAR UMA SEPTAÇÃO ANÔMALA DENTRO DE UM DOS DOIS ÁTRIOS, ALIVIANDO O FLUXO DO SANGUE.	R$ 10.762,50	R$ 5.795,19	R$ 16.557,69	8	Sim	Sim	CORREÇÃO DE ANOMALIAS CARDÍACAS
04.06.01.022-6	CORREÇÃO DE CORONÁRIA ANÔMALA (CRIANÇA E ADOLESCENTE)	01 - Cirurgia cardiovascular	PROCEDIMENTO QUE CONSISTE EM REIMPLANTAR A(S) ARTÉRIA(S) CORONÁRIA(S) NA AORTA, COM OU SEM USO DE RETALHOS OU ENXERTOS TUBULARES.	R$ 14.474,15	R$ 7.793,77	R$ 22.267,92	8	Sim	Sim	PROCEDIMENTOS DE CIRURGIA CARDIOVASCULAR INFANTOJUVENIL
04.06.01.023-4	CORREÇÃO DE DRENAGEM ANÔMALA DO RETORNO SISTÊMICO	01 - Cirurgia cardiovascular	PROCEDIMENTO QUE CONSISTE EM REIMPLANTAR A(S) VEIA(S) CAVA(S) NO ÁTRIO DIREITO, COM OU SEM USO DE RETALHOS OU ENXERTOS TUBULARES.	R$ 6.508,73	R$ 3.365,37	R$ 9.874,10	8	Sim	Sim	CORREÇÃO DE ANOMALIAS CARDÍACAS
04.06.01.024-2	CORREÇÃO DE DRENAGEM ANÔMALA PARCIAL DE VEIAS PULMONARES	01 - Cirurgia cardiovascular	PROCEDIMENTO QUE CONSISTE EM REIMPLANTAR A(S) VEIA(S) PULMONARE(S) NO ÁTRIO ESQUERDO, COM OU SEM USO DE RETALHOS OU ENXERTOS TUBULARES. NÃO INCLUI QUANDO TODAS AS VEIAS PULMONARES ESTÃO COM CONEXÃO ANORMAL.	R$ 6.508,73	R$ 3.365,37	R$ 9.874,10	8	Sim	Sim	CORREÇÃO DE ANOMALIAS CARDÍACAS
04.06.01.025-0	CORREÇÃO DE DRENAGEM ANÔMALA TOTAL DE VEIAS PULMONARES	01 - Cirurgia cardiovascular	PROCEDIMENTO QUE CONSISTE EM REIMPLANTAR TODAS AS VEIAS PULMONARES NO ÁTRIO ESQUERDO, COM OU SEM USO DE RETALHOS OU ENXERTOS TUBULARES.	R$ 15.807,24	R$ 8.511,59	R$ 24.318,83	8	Sim	Sim	CORREÇÃO DE ANOMALIAS CARDÍACAS
04.06.01.026-9	CORREÇÃO DE DUPLA VIA DE SAÍDA DO VENTRÍCULO DIREITO	01 - Cirurgia cardiovascular	PROCEDIMENTO QUE CONSISTE EM TUNELIZAR O SEPTO INTERVENTRICULAR, DE FORMA A DEIXAR O VENTRÍCULO DIREITO CONECTADO A ARTÉRIA PULMONAR AO VENTRÍCULO ESQUERDO CONECTADO A AORTA, COM USO DE RETALHO DE ENXERTO ORGÂNICO E/OU INORGÂNICO.	R$ 15.807,24	R$ 8.511,59	R$ 24.318,83	15	Sim	Sim	CORREÇÃO DE ANOMALIAS CARDÍACAS
04.06.01.027-7	CORREÇÃO DE DUPLA VIA DE SAÍDA DO VENTRÍCULO ESQUERDO	01 - Cirurgia cardiovascular	PROCEDIMENTO QUE CONSISTE EM REIMPLANTAR A(S) VEIA(S) PULMONARE(S) NO ÁTRIO ESQUERDO, COM OU SEM USO DE RETALHOS OU ENXERTOS TUBULARES. NÃO INCLUI QUANDO TODAS AS VEIAS PULMONARES ESTÃO COM CONEXÃO ANORMAL.	R$ 15.807,24	R$ 8.511,59	R$ 24.318,83	8	Sim	Sim	CORREÇÃO DE ANOMALIAS CARDÍACAS
04.06.01.028-5	CORREÇÃO DE ESTENOSE AÓRTICA (0 A 3 ANOS)	01 - Cirurgia cardiovascular	PROCEDIMENTO QUE CONSISTE EM AUMENTAR O FLUXO DE SANGUE DO VENTRÍCULO ESQUERDO PARA A AORTA, TRATANDO A VÁLVULA COM FLUXO RESTRITIVO, SEM PRÓTESE VALVAR.	R$ 13.283,31	R$ 7.152,55	R$ 20.435,86	15	Sim	Sim	PROCEDIMENTOS DE CIRURGIA CARDIOVASCULAR INFANTOJUVENIL
04.06.01.029-3	CORREÇÃO DE ESTENOSE MITRAL CONGÊNITA	01 - Cirurgia cardiovascular	PROCEDIMENTO QUE CONSISTE EM AUMENTAR O FLUXO DE SANGUE DO ÁTRIO ESQUERDO PARA O VENTRÍCULO ESQUERDO, ABRINDO A VÁLVULA COM FLUXO RESTRITIVO, SEM PRÓTESE VALVAR.	R$ 8.528,04	R$ 3.829,47	R$ 12.357,51	8	Sim	Sim	CORREÇÃO DE ANOMALIAS CARDÍACAS
04.06.01.030-7	CORREÇÃO DE ESTENOSE SUPRA-AÓRTICA	01 - Cirurgia cardiovascular	PROCEDIMENTO QUE CONSISTE EM AUMENTAR O FLUXO DE SANGUE DO VENTRÍCULO ESQUERDO PARA A AORTA, TRATANDO A REGIÃO ACIMA DA VALVA, QUE ENCONTRA-SE OBSTRUTIVA, PROMOVENDO AMPLIAÇÃO COM OU SEM RETALHO DE ENXERTO ORGÂNICO OU INORGÂNICO	R$ 4.079,80	R$ 3.365,37	R$ 7.445,17	5	Sim	Sim	CORREÇÃO DE ANOMALIAS CARDÍACAS
04.06.01.031-5	CORREÇÃO DE FÍSTULA AÓRTO-CAVITARIAS	01 - Cirurgia cardiovascular	PROCEDIMENTO QUE CONSISTE EM ELIMINAR COMUNICAÇÕES ANORMAIS ENTRE OS VASOS E CÂMARAS DO CORAÇÃO.	R$ 6.508,73	R$ 3.365,37	R$ 9.874,10	8	Sim	Sim	CORREÇÃO DE ANOMALIAS CARDÍACAS
04.06.01.032-3	CORRECAO DE HIPERTROFIA SEPTAL ASSIMETRICA	01 - Cirurgia cardiovascular	PROCEDIMENTO QUE CONSISTE NA RESSECÇÃO DE UMA FAIXA MUSCULAR NA VIA DE SAÍDA DO VENTRÍCULO ESQUERDO PARA ALIVIAR A OBSTRUÇÃO CAUSADA PELO SEPTO QUE ENCONTRA-SE HIPERTROFIADO, CAUSANDO OBSTRUÇÃO SUBLAVAR AÓRTICA.	R$ 7.544,03	R$ 3.365,37	R$ 10.909,40	8	Sim	Sim	CORREÇÃO DE ANOMALIAS CARDÍACAS
04.06.01.033-1	CORREÇÃO DE HIPOPLASIA DE VENTRÍCULO ESQUERDO	01 - Cirurgia cardiovascular	PROCEDIMENTO QUE CONSISTE EM RECONSTRUIR A AORTA ASCENDENTE E ARCO AÓRTICO, USANDO TECIDO PULMONAR AUTÓLOGO COM OU SEM REMENDO DE ENXERTO ORGÂNICO OU INORGÂNICO	R$ 15.807,13	R$ 8.511,53	R$ 24.318,66	8	Sim	Sim	CORREÇÃO DE ANOMALIAS CARDÍACAS
04.06.01.034-0	CORREÇÃO DE INSUFICIÊNCIA DA VÁLVULA TRICÚSPIDE	01 - Cirurgia cardiovascular	PROCEDIMENTO QUE CONSISTE EM REPARAR VALVA QUE APRESENTA VAZAMENTO, SEM USO DE PRÓTESE VALVAR, MAS PODENDO USAR ANEL PROTÉTICO.	R$ 7.544,03	R$ 3.365,37	R$ 10.909,40	8	Sim	Sim	CORREÇÃO DE ANOMALIAS CARDÍACAS
04.06.01.035-8	CORREÇÃO DE INSUFICIÊNCIA MITRAL CONGÊNITA	01 - Cirurgia cardiovascular	PROCEDIMENTO QUE CONSISTE EM REPARAR VALVA QUE APRESENTA VAZAMENTO, SEM USO DE PRÓTESE VALVAR, MAS PODENDO USAR ANEL PROTÉTICO.	R$ 6.508,63	R$ 3.365,37	R$ 9.874,00	8	Sim	Sim	CORREÇÃO DE ANOMALIAS CARDÍACAS

04.06.01.036-6	CORREÇÃO DE INTERRUPÇÃO DO ARCO AÓRTICO	01 - Cirurgia cardiovascular	PROCEDIMENTO QUE CONSISTE EM RECONSTRUIR O ARCO AÓRTICO E AORTA DESCENDENTE, COM OU SEM ENXERTO TUBULAR ORGÂNICO OU INORGÂNICO, MAIS CORREÇÃO DE COMUNICAÇÃO INTERVENTRICULAR.	R$ 15.807,24	R$ 8.511,59	R$ 24.318,83	8	Sim	Sim	CORREÇÃO DE ANOMALIAS CARDÍACAS
04.06.01.037-4	CORREÇÃO DE JANELA AORTO-PULMONAR (CRIANÇA E ADOLESCENTE)	01 - Cirurgia cardiovascular	PROCEDIMENTO QUE CONSISTE EM INTERROMPER COMUNICAÇÃO ANORMAL ENTRE A AORTA E O TRONCO PULMONAR, ATRAVÉS DE SUTURA DIRETA OU USO DE REMENDO DE ENXERTO ORGÂNICO OU INORGÂNICO	R$ 14.590,27	R$ 7.856,30	R$ 22.446,57	8	Sim	Sim	PROCEDIMENTOS DE CIRURGIA CARDIOVASCULAR INFANTOJUVENIL
04.06.01.038-2	CORREÇÃO DE JANELA AORTO-PULMONAR	01 - Cirurgia cardiovascular	PROCEDIMENTO QUE CONSISTE EM INTERROMPER COMUNICAÇÃO ANORMAL ENTRE A AORTA E O TRONCO PULMONAR, ATRAVÉS DE SUTURA DIRETA OU USO DE REMENDO DE ENXERTO ORGÂNICO OU INORGÂNICO	R$ 7.544,03	R$ 3.365,37	R$ 10.909,40	10	Sim	Sim	CORREÇÃO DE ANOMALIAS CARDÍACAS
04.06.01.039-0	CORREÇÃO DE LESÕES NA TRANSPOSIÇÃO CORRIGIDA DOS VASOS DA BASE	01 - Cirurgia cardiovascular	PROCEDIMENTO QUE CONSISTE EM CORRIGIR DEFEITOS ASSOCIADOS A ESTA DOENÇA, QUE GERALMENTE SÃO INSUFICIÊNCIA TRICÚSPIDE, ESTENOSE PULMONAR, COMUNICAÇÃO INTERVENTRICULAR.	R$ 11.797,80	R$ 6.352,66	R$ 18.150,46	8	Sim	Sim	CORREÇÃO DE ANOMALIAS CARDÍACAS
04.06.01.040-4	CORREÇÃO DE PERSISTÊNCIA DO CANAL ARTERIAL	01 - Cirurgia cardiovascular	PROCEDIMENTO QUE CONSISTE EM INTERROMPER COMUNICAÇÃO ANORMAL ENTRE A AORTA E O TRONCO PULMONAR ATRAVÉS DE SUTURA DIRETA, GERALMENTE SEM USO DE REMENDO DE ENXERTO.	R$ 1.621,75	R$ 1.923,03	R$ 3.544,78	5	Sim	Sim	CORREÇÃO DE ANOMALIAS CARDÍACAS
04.06.01.041-2	CORREÇÃO DE PERSISTÊNCIA DO CANAL ARTERIAL NO RÉCEM-NASCIDO	01 - Cirurgia cardiovascular	PROCEDIMENTO QUE CONSISTE EM INTERROMPER COMUNICAÇÃO ANORMAL ENTRE A AORTA E O TRONCO PULMONAR ATRAVÉS DE SUTURA DIRETA, GERALMENTE SEM USO DE REMENDO DE ENXERTO.	R$ 1.621,75	R$ 1.923,03	R$ 3.544,78	5	Sim	Sim	PROCEDIMENTOS DE CIRURGIA CARDIOVASCULAR INFANTOJUVENIL
04.06.01.042-0	CORREÇÃO DE TETRALOGIA DE FALLOT E VARIANTES (CRIANÇA E ADOLESCENTE)	01 - Cirurgia cardiovascular	PROCEDIMENTO QUE CONSISTE EM CORRIGIR A COMUNICAÇÃO ENTRE OS DOIS VENTRÍCULOS E EM AUMENTAR O FLUXO DE SANGUE ENTRE O VENTRÍCULO DIREITO E AS ARTÉRIAS PULMONARES, ATRAVÉS DE AMPLIAÇÃO E ALARGAMENTO DE PARTE DO VENTRÍCULO DIREITO E/OU ANEL VALVAR PULMONAR E/OU ARTÉRIAS PULMONARES, COM USO DE RETALHOS DE ENXERTOS ORGÂNICOS E/OU INORGÂNICOS COM OU SEM PRÓTESES VALVARES.	R$ 14.590,27	R$ 7.856,30	R$ 22.446,57	15	Sim	Sim	PROCEDIMENTOS DE CIRURGIA CARDIOVASCULAR INFANTOJUVENIL
04.06.01.043-9	CORRECAO DE TETRALOGIA DE FALLOT E VARIANTES	01 - Cirurgia cardiovascular	PROCEDIMENTO QUE CONSISTE EM CORRIGIR A COMUNICAÇÃO ENTRE OS DOIS VENTRÍCULOS E EM AUMENTAR O FLUXO DE SANGUE ENTRE O VENTRÍCULO DIREITO E AS ARTÉRIAS PULMONARES, ATRAVÉS DE AMPLIAÇÃO E ALARGAMENTO DE PARTE DO VENTRÍCULO DIREITO E/OU ANEL VALVAR PULMONAR E/OU ARTÉRIAS PULMONARES, COM USO DE RETALHOS DE ENXERTOS ORGÂNICOS E/OU INORGÂNICOS COM OU SEM PRÓTESES VALVARES. REALIZADO EM PACIENTES ACIMA DE 18 ANOS.	R$ 8.528,04	R$ 3.829,47	R$ 12.357,51	8	Sim	Sim	CORREÇÃO DE ANOMALIAS CARDÍACAS
04.06.01.044-7	CORREÇÃO DE TRANSPOSIÇÃO DOS GRANDES VASOS DA BASE (CRIANÇA E ADOLESCENTE)	01 - Cirurgia cardiovascular	PROCEDIMENTO QUE CONSISTE EM FAZER COM QUE A AORTA RECEBA SANGUE ARTERIAL E A ARTÉRIA PULMONAR RECEBA SANGUE VENOSO. PODE SER FEITO EM NÍVEL ARTERIAL OU EM NÍVEL ATRIAL.	R$ 15.807,24	R$ 8.511,59	R$ 24.318,83	8	Sim	Sim	PROCEDIMENTOS DE CIRURGIA CARDIOVASCULAR INFANTOJUVENIL
04.06.01.045-5	CORREÇÃO DE TRANSPOSIÇÃO DE GRANDES VASOS DA BASE	01 - Cirurgia cardiovascular	PROCEDIMENTO QUE CONSISTE EM FAZER COM QUE A AORTA RECEBA SANGUE ARTERIAL E A ARTÉRIA PULMONAR RECEBA SANGUE VENOSO. PODE SER FEITO A NÍVEL ARTERIAL OU A NÍVEL ATRIAL	R$ 7.544,03	R$ 4.321,19	R$ 11.865,22	8	Sim	Sim	CORREÇÃO DE ANOMALIAS CARDÍACAS
04.06.01.046-3	CORREÇÃO DE TRONCO ARTERIOSO PERSISTENTE	01 - Cirurgia cardiovascular	PROCEDIMENTO QUE CONSISTE EM SEPARAR A AORTA DA ARTÉRIA PULMONAR, RECONSTRUINDO A VIA DE SAÍDA DE VENTRÍCULO DIREITO COM PRÓTESE VALVADA, MAIS CORREÇÃO DE COMUNICAÇÃO INTERVENTRICULAR.	R$ 15.807,24	R$ 8.511,59	R$ 24.318,83	15	Sim	Sim	CORREÇÃO DE ANOMALIAS CARDÍACAS
04.06.01.047-1	CORREÇÃO DE VENTRÍCULO ÚNICO	01 - Cirurgia cardiovascular	A CIRURGIA CONSISTE NA SEPTAÇÃO DO VENTRÍCULO ENTRE AS VÁLVULAS OU NO REDIRECIONAMENTO DO SANGUE NO CORAÇÃO, ANASTOMOSANDO O ÁTRIO DIREITO NA ARTÉRIA PULMONAR (FONTAN). ALGUMAS VEZES É NECESSÁRIO ATUAR SOBRE VÁLVULAS A-V DEFORMADAS OU ATRÉSICAS. FEITA POR TORACOTOMIA COM CIRCULAÇÃO EXTRACORPÓREA.A DOENÇA É DEFINIDA COMO UMA CAVIDADE VENTRICULAR ÚNICA QUE RECEBE DOIS ÁTRIOS COM DUAS VÁLVULAS ÁTRIO-VENTRICULARES.	R$ 15.807,24	R$ 8.511,59	R$ 24.318,83	8	Sim	Sim	CORREÇÃO DE ANOMALIAS CARDÍACAS
04.06.01.048-0	CORREÇÃO DO CANAL ÁTRIO-VENTRICULAR (PARCIAL / INTERMEDIÁRIO)	01 - Cirurgia cardiovascular	PROCEDIMENTO QUE CONSISTE EM CORRIGIR A COMUNICAÇÃO ENTRE OS DOIS VENTRÍCULOS E OS DOIS ÁTRIOS, IMPEDINDO A COMUNICAÇÃO DE SANGUE ENTRE AS CAVIDADES CARDÍACAS, ATRAVÉS DO USO DE RETALHOS DE ENXERTO ORGÂNICO OU INORGÂNICO, MAIS A CORREÇÃO DAS VALVAS QUE SÃO MAL FORMADAS, GERALMENTE SEM USO DE PRÓTESE VALVAR.	R$ 6.508,73	R$ 3.365,37	R$ 9.874,10	8	Sim	Sim	CORREÇÃO DE ANOMALIAS CARDÍACAS
04.06.01.049-8	CORREÇÃO DO CANAL ÁTRIO-VENTRICULAR (TOTAL)	01 - Cirurgia cardiovascular	PROCEDIMENTO QUE CONSISTE EM CORRIGIR A COMUNICAÇÃO ENTRE OS DOIS VENTRÍCULOS E DOS DOIS ÁTRIOS, IMPEDINDO A COMUNICAÇÃO DE SANGUE ENTRE AS CAVIDADES CARDÍACAS, ATRAVÉS DO USO DE RETALHOS DE ENXERTO ORGÂNICO OU INORGÂNICO, MAIS A CORREÇÃO DAS VALVAS QUE SÃO MAL FORMADAS, GERALMENTE SEM USO DE PRÓTESE VALVAR.	R$ 15.807,24	R$ 8.511,59	R$ 24.318,83	8	Sim	Sim	CORREÇÃO DE ANOMALIAS CARDÍACAS
04.06.01.050-1	CORREÇÕES DE ANOMALIAS DO ARCO AÓRTICO	01 - Cirurgia cardiovascular	PROCEDIMENTO QUE CONSISTE EM RESSECAR OU INTERROMPER O FLUXO POR SEGMENTOS EXTRANUMERÁRIOS DE ARCO AÓRTICO OU DE VASOS DA BASE, QUE PODEM CONSTRINGIR ESTRUTURAS VIZINHAS.	R$ 4.190,00	R$ 3.829,47	R$ 8.019,47	5	Sim	Sim	CORREÇÃO DE ANOMALIAS CARDÍACAS
04.06.01.051-0	DRENAGEM C/ BIOPSIA DE PERICÁRDIO	01 - Cirurgia cardiovascular	RESSECÇÃO DE FRAGMENTO DE PERICÁRDIO PARA EXAME DIAGNÓSTICO E DRENAGEM DESCOMPRESSIVA DO LÍQUIDO PERICÁRDICO.	R$ 56,93	R$ 212,82	R$ 269,75	3	Sim	Sim	CARDIORRESSECÇÃO

04.06.01.052-8	EXERESE DE CISTO PERICÁRDICO	01 - Cirurgia cardiovascular	RETIRADA DE TUMOR BENIGNO PARA DIAGNÓSTICO E/OU DESCOMPRESSÃO PERICÁRDICA.	R$ 2.879,75	R$ 1.085,74	R$ 3.965,49	5	Sim	Sim	CARDIORRESSECÇÃO
04.06.01.069-2	IMPLANTE DE PRÓTESE VALVAR	01 - Cirurgia cardiovascular	SUBSTITUIÇÃO DA VÁLVULA CARDÍACA ORIGINAL DOENTE POR UMA PRÓTESE BIOLÓGICA OU METÁLICA, REALIZADA POR TORACOTOMIA E COM CIRCULAÇÃO EXTRACORPÓREA.	R$ 2.956,37	R$ 4.584,31	R$ 7.540,68	5	Sim	Sim	OUTROS PROCEDIMENTOS CIRÚRGICOS CARDIOVASCULARES
04.06.01.071-4	INSTALAÇÃO DE ASSISTÊNCIA CIRCULATÓRIA	01 - Cirurgia cardiovascular	IMPLANTAÇÃO DE DISPOSITIVO PARA AJUDAR O/S VENTÍCULO/S SE RECUPERAR/EM. INDICADO PARA AGUARDAR DOADOR PARA TRANSPLANTE CARDÍACO RECUPERAR APÓS INFARTOS DO MIOCÁRDIO AGUDOS COM GRAVE DISFUNÇÃO DO CORAÇÃO.	R$ 49,50	R$ 295,16	R$ 344,66	2	Sim	Sim	OUTROS PROCEDIMENTOS CIRÚRGICOS CARDIOVASCULARES
04.06.01.055-2	IMPLANTE C/ TROCA DE POSIÇÃO DE VALVAS (CIRURGIA DE ROSS)	01 - Cirurgia cardiovascular	PROCEDIMENTO QUE CONSISTE EM RESSECAR A VALVA E O TRONCO PULMONAR DO PACIENTE E IMPLANTAR NO LOCAL DA AORTA, MAIS O IMPLANTE DE UM ENXERTO ORGÂNICO (HOMOENXERTO) NA POSIÇÃO PULMONAR.	R$ 3.248,03	R$ 4.321,19	R$ 7.569,22	5	Sim	Sim	CARDIORRESSECÇÃO
04.06.01.056-0	IMPLANTE DE CARDIOVERSOR DESFIBRILADOR DE CÂMARA ÚNICA TRANSVENOSO	01 - Cirurgia cardiovascular	IMPLANTE DE DISPOSITIVO ELETRÔNICO DE CÂMARA ÚNICA PARA TRATAMENTO DAS TAQUIARRITMIAS COM COLOCAÇÃO DE ELETRODO POR VIA ENDOVENOSA.	R$ 868,71	R$ 1.075,03	R$ 1.943,74	2	Sim	Não	IMPLANTE DE DCEI
04.06.01.057-9	IMPLANTE DE CARDIOVERSOR DESFIBRILADOR (CDI) MULTI-SITIO TRANSVENOSO EPIMIOCÁRDICO POR TORACOTOMIA P/ IMPLANTE DE ELETRODO	01 - Cirurgia cardiovascular	IMPLANTE DE DISPOSITIVO ELETRÔNICO PARA TRATAMENTO DAS TAQUIARRITMIAS E DA INSUFICIÊNCIA CARDÍACA COM COLOCAÇÃO DE ELETRODOS POR VIA ENDOVENOSA E EPIMIOCÁRDICA.	R$ 1.192,51	R$ 1.173,94	R$ 2.366,45	5	Sim	Sim	IMPLANTE DE DCEI
04.06.01.058-7	IMPLANTE DE CARDIOVERSOR DESFIBRILADOR DE CÃMARA DUPLA TRANSVENOSO	01 - Cirurgia cardiovascular	IMPLANTE DE DISPOSITIVO ELETRÔNICO DE CÂMARA DUPLA PARA TRATAMENTO DAS TAQUIARRITMIAS COM COLOCAÇÃO DE ELETRODOS POR VIA ENDOVENOSA.	R$ 854,96	R$ 1.173,94	R$ 2.028,90	2	Sim	Sim	IMPLANTE DE DCEI
04.06.01.059-5	IMPLANTE DE CARDIOVERSOR DESFIBRILADOR MULTI-SÍTIO ENDOCAVITÁRIO C/ REVERSÃO PARA EPIMIOCÁRDICO POR TORACOTOMIA	01 - Cirurgia cardiovascular	IMPLANTE DE DISPOSITIVO ELETRÔNICO PARA TRATAMENTO DAS TAQUIARRITMIAS E DA INSUFICIÊNCIA CARDÍACA COM COLOCAÇÃO DE ELETRODOS POR VIA ENDOVENOSA, COM REVERSÃO DO IMPLANTE DE ELETRODO DE VENTRÍCULO ESQUERDO PARA EPIMIOCÁRDICO.	R$ 1.175,18	R$ 1.173,94	R$ 2.349,12	5	Sim	Sim	IMPLANTE DE DCEI
04.06.01.060-9	IMPLANTE DE CARDIOVERSOR DESFIBRILADOR (CDI) MULTI-SITIO TRANSVENOSO	01 - Cirurgia cardiovascular	IMPLANTE DE DISPOSITIVO ELETRÔNICO PARA TRATAMENTO DAS TAQUIARRITMIAS E DA INSUFICIÊNCIA CARDÍACA COM COLOCAÇÃO DE ELETRODOS POR VIA ENDOVENOSA.	R$ 868,71	R$ 1.075,03	R$ 1.943,74	3	Sim	Sim	IMPLANTE DE DCEI
04.06.01.061-7	IMPLANTE DE MARCAPASSO CARDÍACO MULTI-SITIO ENDOCAVITÁRIO C/ REVERSÃO P/ EPIMIOCÁRDICO (POR TORACOTOMIA)	01 - Cirurgia cardiovascular	IMPLANTE DE DISPOSITIVO ELETRÔNICO PARA TRATAMENTO DA INSUFICIÊNCIA CARDÍACA COM COLOCAÇÃO DE ELETRODOS POR VIA ENDOVENOSA, COM REVERSÃO DO IMPLANTE DE ELETRODO DE VENTRÍCULO ESQUERDO PARA EPIMIOCÁRDICO.	R$ 1.175,18	R$ 835,79	R$ 2.010,97	5	Sim	Sim	IMPLANTE DE DCEI
04.06.01.062-5	IMPLANTE DE MARCAPASSO CARDÍACO MULTI-SITIO EPIMIOCÁRDICO POR TORACOTOMIA P/IMPLANTE DE ELETRODO	01 - Cirurgia cardiovascular	IMPLANTE DE DISPOSITIVO ELETRÔNICO PARA TRATAMENTO DA INSUFICIÊNCIA CARDÍACA COM COLOCAÇÃO DE ELETRODOS EPIMIOCÁRDICOS.	R$ 1.192,51	R$ 835,79	R$ 2.028,30	5	Sim	Sim	IMPLANTE DE DCEI
04.06.01.063-3	IMPLANTE DE MARCAPASSO CARDÍACO MULTI-SITIO TRANSVENOSO	01 - Cirurgia cardiovascular	IMPLANTE DE DISPOSITIVO ELETRÔNICO PARA TRATAMENTO DA INSUFICIÊNCIA CARDÍACA COM COLOCAÇÃO DE ELETRODOS POR VIA ENDOVENOSA.	R$ 868,71	R$ 1.173,94	R$ 2.042,65	2	Sim	Sim	IMPLANTE DE DCEI
04.06.01.064-1	IMPLANTE DE MARCAPASSO DE CÂMARA DUPLA EPIMIOCÁRDICO	01 - Cirurgia cardiovascular	IMPLANTE DE DISPOSITIVO ELETRÔNICO PARA TRATAMENTO DAS BRADIARRITMIAS CARDÍACAS COM COLOCAÇÃO DE ELETRODOS EPIMIOCÁRDICOS.	R$ 301,60	R$ 669,59	R$ 971,19	2	Sim	Sim	IMPLANTE DE DCEI
04.06.01.065-0	IMPLANTE DE MARCAPASSO DE CÂMARA DUPLA TRANSVENOSO	01 - Cirurgia cardiovascular	IMPLANTE DE DISPOSITIVO ELETRÔNICO PARA TRATAMENTO DAS BRADIARRITMIAS CARDÍACAS COM COLOCAÇÃO DE ELETRODOS ENDOVENOSOS.	R$ 466,24	R$ 759,40	R$ 1.225,64	2	Sim	Sim	IMPLANTE DE DCEI

04.06.01.066-8	IMPLANTE DE MARCAPASSO DE CÂMARA ÚNICA EPIMIOCÁRDICO	01 - Cirurgia cardiovascular	IMPLANTE DE DISPOSITIVO ELETRÔNICO PARA TRATAMENTO DAS BRADIARRITMIAS CARDÍACAS COM COLOCAÇÃO DE ELETRODO EPIMIOCÁRDICO.	R$ 305,20	R$ 669,59	R$ 974,79	2	Sim	Sim	IMPLANTE DE DCEI
04.06.01.067-6	IMPLANTE DE MARCAPASSO DE CÂMARA ÚNICA TRANSVENOSO	01 - Cirurgia cardiovascular	IMPLANTE DE DISPOSITIVO ELETRÔNICO PARA TRATAMENTO DAS BRADIARRITMIAS CARDÍACAS COM COLOCAÇÃO DE ELETRODO ENDOVENOSO.	R$ 466,24	R$ 684,55	R$ 1.150,79	2	Sim	Sim	IMPLANTE DE DCEI
04.06.01.068-4	IMPLANTE DE MARCAPASSO TEMPORÁRIO TRANSVENOSO	01 - Cirurgia cardiovascular	IMPLANTE DE ELETRODO TEMPORÁRIO VIA ENDOVENOSA CONECTADO À MARCAPASSO EXTERNO.	R$ 126,39	R$ 238,36	R$ 364,75	2	Sim	Sim	IMPLANTE DE DCEI
04.06.01.077-3	PERICARDIOCENTESE	01 - Cirurgia cardiovascular	CONSISTE NO PROCESSO DE RETIRADA DE LÍQUIDO ACUMULADO NA MEMBRANA QUE ENVOLVE O CORAÇÃO ATRAVÉS DA INTRODUÇÃO DE UMA SERINGA DE MÉDIO PARA GRANDE CALIBRE POUCO ABAIXO DO ESTERNO E POUCO A ESQUERDA DO TÓRAX COM PEQUENA INCLINAÇÃO VOLTANDO A AGULHA PARA O BRAÇO ESQUERDO DO PACIENTE NO ÂNGULO INFRA-ESTERNAL, SUBXIFOIDE OU AINDA PARA-ESTERNAL.	R$ 49,50	R$ 193,47	R$ 242,97	3	Sim	Não	OUTROS PROCEDIMENTOS CIRÚRGICOS CARDIOVASCULARES
04.06.01.091-9	RETIRADA DE SISTEMA DE ESTIMULAÇÃO CARDÍACA ARTIFICIAL	01 - Cirurgia cardiovascular	RETIRADA DE DISPOSITIVO ELÉTRICO IMPLANTÁVEL.	R$ 271,77	R$ 296,43	R$ 568,20	2	Sim	Não	OUTROS PROCEDIMENTOS CIRÚRGICOS CARDIOVASCULARES
04.06.01.096-0	TRATAMENTO DE CONTUSÃO MIOCÁRDICA	01 - Cirurgia cardiovascular	CIRURGIA PARA SUTURAR O CORAÇÃO QUE FOI SUBMETIDO A UM TRAUMA. PODE OU NÃO NECESSITAR DE CIRCULAÇÃO EXTRACORPÓREA.	R$ 111,75	R$ 44,53	R$ 156,28	4	Sim	Não	OUTROS PROCEDIMENTOS CIRÚRGICOS CARDIOVASCULARES
04.06.01.072-2	INSTALAÇÃO DE CATETER DE TERMODILUIÇÃO	01 - Cirurgia cardiovascular	PARA AVALIAR A FUNÇÃO DAS CÂMARAS CARDÍACAS MEDINDO O DÉBITO CARDÍACO ATRAVÉS DA TERMODILUIÇÃO.	R$ 20,00	R$ 0,00	R$ 20,00	-	Não	Não	CATETERISMO
04.06.01.097-8	TRATAMENTO DE FERIMENTO CARDÍACO PERFURO-CORTANTE	01 - Cirurgia cardiovascular	CIRURGIA PARA SUTURAR O CORAÇÃO QUE FOI FERIDO POR INSTRUMENTO PERFURO-CORTANTE.	R$ 111,75	R$ 44,53	R$ 156,28	2	Sim	Não	OUTROS PROCEDIMENTOS CIRÚRGICOS CARDIOVASCULARES
04.06.01.074-9	MANUTENÇÃO DE ASSISTÊNCIA CIRCULATÓRIA	01 - Cirurgia cardiovascular	MANUTENÇÃO DO FUNCIONAMENTO DE SISTEMA DE SUPORTE CIRCULATÓRIO.O USO DE DISPOSITIVOS DE ASSISTÊNCIA CIRCULATÓRIA É UTILIZADO EM QUADROS GRAVES DE FALÊNCIA HEMODINÂMICA, POIS PODE SER EFETIVO NA RECUPERAÇÃO DA FUNÇÃO CARDÍACA. A LITERATURA MENCIONA CONDIÇÕES EM QUE O SUPORTE CIRCULATÓRIO PODE SER UTILIZADO: MIOCARDITES FULMINANTES; CARDIOMIOPATIAS DILATADAS IDIOPÁTICAS; ARRITMIAS VENTRICULARES RECORRENTES NÃO RESPONSIVAS, PÓS PERICARDIOTOMIA, PÓS INFARTO AGUDO DO MIOCÁRDIO, PODENDO APRESENTAR MELHORA DOS PARÂMETROS HEMODINÂMICOS.	R$ 49,50	R$ 188,91	R$ 238,41	-	Não	Não	TROCA E/OU MANUTENÇÃO DE DISPOSITIVOS CARDÍACOS IMPLANTÁVEIS
04.06.01.075-7	PERICARDIECTOMIA	01 - Cirurgia cardiovascular	RESSECÇÃO DA MAIOR PARTE DO PERICÁRDIO DOENTE, QUE RESTRINGE O FUNCIONAMENTO DO CORAÇÃO.	R$ 1.144,52	R$ 995,22	R$ 2.139,74	5	Sim	Sim	CARDIORRESSECÇÃO
04.06.01.076-5	PERICARDIECTOMIA PARCIAL	01 - Cirurgia cardiovascular	RESSECÇÃO DE FRAGMENTO DO PERICÁRDIO PARA MELHORAR A FUNÇÃO DO CORAÇÃO.	R$ 1.144,52	R$ 995,22	R$ 2.139,74	5	Sim	Sim	CARDIORRESSECÇÃO
04.06.01.098-6	TROCA DE AORTA ASCENDENTE	01 - Cirurgia cardiovascular	CONSISTE NA SUBSTITUIÇÃO DA AORTA ASCENDENTE POR UM ENXERTO TUBULAR, ORGÂNICO OU INORGÂNICO. REALIZADA POR TORACOTOMIA E COM CIRCULAÇÃO EXTRACORPÓREA.	R$ 2.956,37	R$ 5.718,97	R$ 8.675,34	5	Sim	Sim	OUTROS PROCEDIMENTOS CIRÚRGICOS CARDIOVASCULARES
04.06.01.078-1	PLÁSTICA / TROCA DE VÁLVULA TRICÚSPIDE (ANOMALIA DE EBSTEIN)	01 - Cirurgia cardiovascular	CONSISTE EM REPARAR VALVA ENTRE O ÁTRIO DIREITO E O VENTRÍCULO DIREITO, CONGENITAMENTE MAL FORMADA, QUE APRESENTA VAZAMENTO, COM OU SEM USO DE PRÓTESE VALVAR OU ANEL PROTÉTICO, NA ANOMALIA DE EBSTEIN.	R$ 15.807,24	R$ 8.511,59	R$ 24.318,83	15	Sim	Sim	PLÁSTICA E/OU ENXERTOS CARDÍACOS
04.06.01.079-0	PLÁSTICA DE LOJA DE GERADOR DE SISTEMA DE ESTIMULAÇÃO CARDÍACA ARTIFICIAL	01 - Cirurgia cardiovascular	CIRURGIA PARA RECONSTRUIR BOLSA DO GERADOR DE MARCAPASSO DE FORMA QUE ESTE VOLTE A FUNCIONAR NORMALMENTE.	R$ 271,77	R$ 538,38	R$ 810,15	2	Sim	Não	PLÁSTICA E/OU ENXERTOS CARDÍACOS
04.06.01.080-3	PLÁSTICA VALVAR	01 - Cirurgia cardiovascular	CONSISTE EM RESTABELECER O FUNCIONAMENTO NORMAL DAS VÁLVULAS CARDÍACAS SEM O USO DE UMA PRÓTESE, PODENDO UTILIZAR UM ANEL DE SUSTENTAÇÃO. REALIZADA POR TORACOTOMIA E COM CIRCULAÇÃO EXTRACORPÓREA.	R$ 2.824,37	R$ 4.409,89	R$ 7.234,26	5	Sim	Sim	PLÁSTICA E/OU ENXERTOS CARDÍACOS
04.06.01.081-1	PLÁSTICA VALVAR C/ REVASCULARIZAÇÃO MIOCÁRDICA	01 - Cirurgia cardiovascular	CIRURGIA DE RECONSTRUÇÃO DA VÁLVULA CARDÍACA, SEM O USO DE PRÓTESE VALVULAR, PORÉM PODENDO USAR UM ANEL DE SUSTENTAÇÃO, IMPLANTANDO SIMULTANEAMENTE PONTES ARTERIAIS E/OU VENOSAS NA/S ARTÉRIA/S CORONÁRIA/S. REALIZADA POR TORACOTOMIA E COM CIRCULAÇÃO EXTRACORPÓREA.	R$ 2.956,37	R$ 6.538,56	R$ 9.494,93	5	Sim	Sim	PLÁSTICA E/OU ENXERTOS CARDÍACOS

04.06.01.082-0	PLÁSTICA VALVAR E/OU TROCA VALVAR MÚLTIPLA	01 - Cirurgia cardiovascular	CIRURGIA COM PLÁSTICA OU TROCA DE MAIS DE UMA VÁLVULA CARDÍACA (MITRAL, AÓRTICA, TRICÚSPIDE OU PULMONAR) COM PRÓTESE BIOLÓGICA OU METÁLICA. REALIZADA POR TORACOTOMIA E COM CIRCULAÇÃO EXTRACORPÓREA.	R$ 2.956,37	R$ 5.886,28	R$ 8.842,65	5	Sim	Sim	PLÁSTICA E/OU ENXERTOS CARDÍACOS
04.06.01.083-8	RECONSTRUÇÃO DA RAIZ DA AORTA	01 - Cirurgia cardiovascular	CIRURGIA QUE CONSISTE NA SUBSTITUIÇÃO DA RAIZ DA AORTA POR UM ENXERTO TUBULAR NÃO VALVADO, ORGÂNICO OU INORGÂNICO. REALIZADA POR TORACOTOMIA E COM CIRCULAÇÃO EXTRACORPÓREA	R$ 2.983,09	R$ 7.132,96	R$ 10.116,05	5	Sim	Sim	PLÁSTICA E/OU ENXERTOS CARDÍACOS
04.06.01.084-6	RECONSTRUÇÃO DA RAIZ DA AORTA C/ TUBO VALVADO	01 - Cirurgia cardiovascular	CIRURGIA QUE CONSISTE NA SUBSTITUIÇÃO DA RAIZ DA AORTA POR UM ENXERTO TUBULAR VALVADO, ORGÂNICO OU INORGÂNICO, COM REIMPLANTE DAS ARTÉRIAS CORONÁRIAS. REALIZADA POR TORACOTOMIA E COM CIRCULAÇÃO EXTRACORPÓREA.	R$ 2.983,09	R$ 8.155,20	R$ 11.138,29	5	Sim	Sim	PLÁSTICA E/OU ENXERTOS CARDÍACOS
04.06.01.085-4	REPOSICIONAMENTO DE ELETRODOS DE CARDIOVERSOR DESFIBRILADOR	01 - Cirurgia cardiovascular	RECOLOCAÇÃO DE ELETRODOS DESLOCADOS DE CARDIODESFIBRILADOR.	R$ 271,77	R$ 626,35	R$ 898,12	2	Sim	Não	TROCA E/OU MANUTENÇÃO DE DISPOSITIVOS CARDÍACOS IMPLANTÁVEIS
04.06.01.086-2	REPOSICIONAMENTO DE ELETRODOS DE MARCAPASSO	01 - Cirurgia cardiovascular	RECOLOCAÇÃO DE ELETRODOS DESLOCADOS DE MARCAPASSO.	R$ 271,77	R$ 644,51	R$ 916,28	2	Sim	Não	TROCA E/OU MANUTENÇÃO DE DISPOSITIVOS CARDÍACOS IMPLANTÁVEIS
04.06.01.087-0	REPOSICIONAMENTO DE ELETRODOS DE MARCAPASSO MULTI-SITIO	01 - Cirurgia cardiovascular	RECOLOCAÇÃO DE ELETRODOS DESLOCADOS DE MARCAPASSO MULTI-SÍTIO.	R$ 271,77	R$ 626,35	R$ 898,12	2	Sim	Não	TROCA E/OU MANUTENÇÃO DE DISPOSITIVOS CARDÍACOS IMPLANTÁVEIS
04.06.01.088-9	RESSECÇÃO DE ENDOMIOCARDIOFIBROSE	01 - Cirurgia cardiovascular	CONSISTE EM RESSECAR TECIDO ANÔMALO QUE RECOBRE AS PAREDES INTERNAS DAS CÂMARAS DO CORAÇÃO E PREJUDICA O FUNCIONAMENTO CARDÍACO.	R$ 3.110,03	R$ 3.943,72	R$ 7.053,75	5	Sim	Sim	CARDIORRESSECÇÃO
04.06.01.089-7	RESSECÇÃO DE MEMBRANA SUB-AÓRTICA	01 - Cirurgia cardiovascular	CONSISTE EM RESSECAR TECIDO ANÔMALO POSICIONADO ABAIXO DA VALVA AÓRTICA E QUE PREJUDICA O FLUXO DE SANGUE.	R$ 4.079,80	R$ 3.943,72	R$ 8.023,52	5	Sim	Sim	CARDIORRESSECÇÃO
04.06.01.090-0	RESSECÇÃO DE TUMOR INTRACARDÍACO	01 - Cirurgia cardiovascular	CONSISTE EM EXTIRPAR TUMOR DENTRO DAS CÂMARAS CARDÍACAS.	R$ 7.544,03	R$ 4.584,31	R$ 12.128,34	8	Sim	Sim	CARDIORRESSECÇÃO
04.06.01.099-4	TROCA DE ARCO AÓRTICO	01 - Cirurgia cardiovascular	TROCA DA CROÇA DA AORTA POR UM TUBO DE MATERIAL ORGÂNICO OU SINTÉTICO, COM O REIMPLANTE DO TRONCO ARTERIAL BRAQUIOCEFÁLICO E DAS ARTÉRIAS CARÓTIDA E SUBCLÁVIA ESQUERDAS. REALIZADA POR TORACOTOMIA E COM CIRCULAÇÃO EXTRACORPÓREA.	R$ 2.956,37	R$ 5.718,97	R$ 8.675,34	5	Sim	Sim	OUTROS PROCEDIMENTOS CIRÚRGICOS CARDIOVASCULARES
04.06.01.092-7	REVASCULARIZAÇÃO MIOCÁRDICA C/ USO DE EXTRACÓRPOREA	01 - Cirurgia cardiovascular	IMPLANTES DE PONTE/S DE ARTÉRIA/S E/OU VEIA/S, PARA PERFUNDIR MELHOR O CORAÇÃO. REALIZADA POR TORACOTOMIA E COM CIRCULAÇÃO EXTRACORPÓREA. ESTÁ INCLUÍDO NO PROCEDIMENTO A RETIRADA DOS ENXERTOS.	R$ 2.956,37	R$ 5.176,36	R$ 8.132,73	5	Sim	Sim	REVASCULARIZAÇÃO MIOCÁRDICA
04.06.01.093-5	REVASCULARIZAÇÃO MIOCÁRDICA C/ USO DE EXTRACÓRPOREA (C/ 2 OU MAIS ENXERTOS)	01 - Cirurgia cardiovascular	IMPLANTES DE PONTE/S DE ARTÉRIA/S E/OU VEIA/S, PARA PERFUNDIR MELHOR O CORAÇÃO. REALIZADA POR TORACOTOMIA E COM CIRCULAÇÃO EXTRACORPÓREA. ESTÁ INCLUÍDO NO PROCEDIMENTO A RETIRADA DOS ENXERTOS.	R$ 2.956,37	R$ 5.448,80	R$ 8.405,17	5	Sim	Sim	REVASCULARIZAÇÃO MIOCÁRDICA
04.06.01.094-3	REVASCULARIZAÇÃO MIOCÁRDICA S/ USO DE EXTRACORPÓREA	01 - Cirurgia cardiovascular	IMPLANTES DE PONTE/S DE ARTÉRIA/S E/OU VEIA/S, PARA PERFUNDIR MELHOR O CORAÇÃO. REALIZADA POR TORACOTOMIA E SEM CIRCULAÇÃO EXTRACORPÓREA. ESTÁ INCLUÍDO NO PROCEDIMENTO A RETIRADA DOS ENXERTOS.	R$ 4.940,43	R$ 5.176,36	R$ 10.116,79	5	Sim	Sim	REVASCULARIZAÇÃO MIOCÁRDICA
04.06.01.095-1	REVASCULARIZAÇÃO MIOCÁRDICA S/ USO DE EXTRACORPÓREA (C/ 2 OU MAIS ENXERTOS)	01 - Cirurgia cardiovascular	IMPLANTES DE PONTE/S DE ARTÉRIA/S E/OU VEIA/S, PARA PERFUNDIR MELHOR O CORAÇÃO. REALIZADA POR TORACOTOMIA E SEM CIRCULAÇÃO EXTRACORPÓREA. ESTÁ INCLUÍDO NO PROCEDIMENTO A RETIRADA DOS ENXERTOS.	R$ 4.940,43	R$ 5.176,36	R$ 10.116,79	5	Sim	Sim	REVASCULARIZAÇÃO MIOCÁRDICA
04.06.01.120-6	TROCA VALVAR C/ REVASCULARIZAÇÃO MIOCÁRDICA	01 - Cirurgia cardiovascular	TROCA DA VÁLVULA MITRAL OU AÓRTICA POR PRÓTESE BIOLÓGICA OU METÁLICA E IMPLANTE DE PONTE/S DE VEIA/S SAFENA/S E/OU ARTÉRIA/S MAMÁRIA/S NA/S ARTÉRIA/S CORONÁRIA/S PARA MELHORAR A PERFUSÃO DE SANGUE AO MIOCÁRDIO. REALIZADA POR TORACOTOMIA MEDIANA TRANSESTERNAL E PERICARDIOTOMIA LONGITUDINAL E SAFENECTOMIA E COM CIRCULAÇÃO EXTRACORPÓREA.	R$ 2.956,37	R$ 6.538,56	R$ 9.494,93	5	Sim	Sim	OUTROS PROCEDIMENTOS CIRÚRGICOS CARDIOVASCULARES
04.06.01.009-9	BANDAGEM DA ARTÉRIA PULMONAR	01 - Cirurgia cardiovascular	PROCEDIMENTO QUE CONSISTE EM DIMINUIR O CALIBRE DA(S) ARTÉRIA(S) PULMONAR(ES) COM USO DE DISPOSITIVOS OU RETALHOS DE ENXERTO AO REDOR DO VASO PARA DIMINUIR O FLUXO DE SANGUE PARA O PULMÃO E/OU AUMENTAR A PRESSÃO NO VENTRÍCULO SUBPULMONAR.	R$ 3.706,55	R$ 1.923,03	R$ 5.629,58	5	Sim	Sim	OUTROS PROCEDIMENTOS CARDIOVASCULARES DE CORREÇÃO
04.06.01.053-6	FECHAMENTO DE COMUNICAÇÃO INTERATRIAL	01 - Cirurgia cardiovascular	PROCEDIMENTO QUE CONSISTE EM CORRIGIR A COMUNICAÇÃO ENTRE OS DOIS ÁTRIOS, IMPEDINDO A COMUNICAÇÃO DE SANGUE ENTRE ELES ATRAVÉS DO USO DE RETALHOS DE ENXERTO ORGÂNICO OU INORGÂNICO.	R$ 4.079,80	R$ 3.365,37	R$ 7.445,17	5	Sim	Sim	OUTROS PROCEDIMENTOS CARDIOVASCULARES DE CORREÇÃO

04.06.01.100-1	TROCA DE CONJUNTO DO SEIO CORONÁRIO NO MARCAPASSO MULTI-SITIO	01 - Cirurgia cardiovascular	TROCA DE SISTEMA DE ELETRODOS DO SEIO CORONARIANO MALFUNCIONANTE EM MARCAPASSO MULTI-SÍTIO.	R$ 271,77	R$ 716,29	R$ 988,06	2	Sim	Não	TROCA E/OU MANUTENÇÃO DE DISPOSITIVOS CARDÍACOS IMPLANTÁVEIS
04.06.01.101-0	TROCA DE ELETRODOS DE DESFIBRILADOR DE CARDIO-DESFIBRILADOR TRANSVENOSO	01 - Cirurgia cardiovascular	TROCA DE ELETRODOS MALFUNCIONANTES DE DESFIBRILADOR.	R$ 271,77	R$ 626,35	R$ 898,12	2	Sim	Não	TROCA E/OU MANUTENÇÃO DE DISPOSITIVOS CARDÍACOS IMPLANTÁVEIS
04.06.01.102-8	TROCA DE ELETRODOS DE DESFIBRILADOR NO CARDIO-DESFIBRILADOR MULTI-SITIO	01 - Cirurgia cardiovascular	TROCA DE ELETRODOS DE DESFIBRILADOR MALFUNCIONANTES EM CARDIO-DESFIBRILADOR MULTI-SÍTIO.	R$ 271,77	R$ 626,35	R$ 898,12	2	Sim	Não	TROCA E/OU MANUTENÇÃO DE DISPOSITIVOS CARDÍACOS IMPLANTÁVEIS
04.06.01.103-6	TROCA DE ELETRODOS DE MARCAPASSO DE CÂMARA DUPLA	01 - Cirurgia cardiovascular	TROCA DE ELETRODOS MALFUNCIONANTES EM MARCAPASSO DE DUPLA CÂMARA.	R$ 304,77	R$ 644,51	R$ 949,28	2	Sim	Não	TROCA E/OU MANUTENÇÃO DE DISPOSITIVOS CARDÍACOS IMPLANTÁVEIS
04.06.01.104-4	TROCA DE ELETRODOS DE MARCAPASSO DE CÂMARA ÚNICA	01 - Cirurgia cardiovascular	TROCA DE ELETRODOS MALFUNCIONANTES EM MARCAPASSO DE CÂMARA ÚNICA.	R$ 271,77	R$ 644,51	R$ 916,28	2	Sim	Não	TROCA E/OU MANUTENÇÃO DE DISPOSITIVOS CARDÍACOS IMPLANTÁVEIS
04.06.01.105-2	TROCA DE ELETRODOS DE MARCAPASSO EM CARDIO-DESFIBRILADOR DE CAMARA DUPLA TRANSVENOSO	01 - Cirurgia cardiovascular	TROCA DE ELETRODO DE MARCAPASSO MALFUNCIONANTE EM CARDIODESFIBRILADOR DE CÂMARA DUPLA TRANSVENOSO.	R$ 82,95	R$ 626,35	R$ 709,30	2	Sim	Não	TROCA E/OU MANUTENÇÃO DE DISPOSITIVOS CARDÍACOS IMPLANTÁVEIS
04.06.01.107-9	TROCA DE ELETRODOS DE MARCAPASSO NO CARDIO-DESFIBRILADOR MULTI-SITIO	01 - Cirurgia cardiovascular	TROCA DE ELETRODO DE MARCAPASSO NO CARDIODESFIBRILADOR MULTI-SITIO	R$ 271,77	R$ 626,35	R$ 898,12	2	Sim	Não	TROCA E/OU MANUTENÇÃO DE DISPOSITIVOS CARDÍACOS IMPLANTÁVEIS
04.06.01.108-7	TROCA DE ELETRODOS DE MARCAPASSO NO MARCAPASSO MULTI-SITIO	01 - Cirurgia cardiovascular	TROCA DE ELETRODO DE MARCAPASSO NO MARCAPASSO MULTI-SITIO	R$ 271,77	R$ 573,58	R$ 845,35	2	Sim	Não	TROCA E/OU MANUTENÇÃO DE DISPOSITIVOS CARDÍACOS IMPLANTÁVEIS
04.06.01.109-5	TROCA DE ELETRODOS DE SEIO CORONÁRIO NO CARDIOVERSOR DESFIBRILADOR MULTI-SITIO	01 - Cirurgia cardiovascular	TROCA DE SISTEMA DE ELETRODOS DE SEIO CORONÁRIO MALFUNCIONANTE EM CARDIO-DESFIBRILADOR MULTI-SÍTIO.	R$ 271,77	R$ 573,58	R$ 845,35	1	Sim	Não	TROCA E/OU MANUTENÇÃO DE DISPOSITIVOS CARDÍACOS IMPLANTÁVEIS
04.06.01.110-9	TROCA DE GERADOR DE CARDIO-DESFIBRILADOR DE CÂMARA ÚNICA / DUPLA	01 - Cirurgia cardiovascular	TROCA DE GERADOR DE CARDIO-DESFIBRILADOR MALFUNCIONANTE.	R$ 271,77	R$ 626,35	R$ 898,12	2	Sim	Não	TROCA E/OU MANUTENÇÃO DE DISPOSITIVOS CARDÍACOS IMPLANTÁVEIS
04.06.01.111-7	TROCA DE GERADOR DE CARDIO-DESFIBRILADOR MULTI-SITIO	01 - Cirurgia cardiovascular	TROCA DE GERADOR DE CARDIO-DESFIBRILADOR MULTI-SÍTIO MALFUNCIONANTE.	R$ 271,77	R$ 626,35	R$ 898,12	2	Sim	Não	TROCA E/OU MANUTENÇÃO DE DISPOSITIVOS CARDÍACOS IMPLANTÁVEIS
04.06.01.112-5	TROCA DE GERADOR DE MARCAPASSO DE CÂMARA DUPLA	01 - Cirurgia cardiovascular	TROCA DE GERADOR DE MARCAPASSO DE CÂMARA DUPLA MALFUNCIONANTE.	R$ 271,77	R$ 644,84	R$ 916,61	2	Sim	Não	TROCA E/OU MANUTENÇÃO DE DISPOSITIVOS CARDÍACOS IMPLANTÁVEIS
04.06.01.113-3	TROCA DE GERADOR DE MARCAPASSO DE CÂMARA ÚNICA	01 - Cirurgia cardiovascular	TROCA DE GERADOR DE MARCAPASSO DE CÂMARA ÚNICA MALFUNCIONANTE.	R$ 271,77	R$ 644,51	R$ 916,28	2	Sim	Não	TROCA E/OU MANUTENÇÃO DE DISPOSITIVOS CARDÍACOS IMPLANTÁVEIS

04.06.01.114-1	TROCA DE GERADOR DE MARCAPASSO MULTI-SITIO	01 - Cirurgia cardiovascular	TROCA DE GERADOR DE MARCAPASSO MULTI-SÍTIO MALFUNCIONANTE.	R$ 271,77	R$ 626,35	R$ 898,12	2	Sim	Não	TROCA E/OU MANUTENÇÃO DE DISPOSITIVOS CARDÍACOS IMPLANTÁVEIS
04.06.01.115-0	TROCA DE GERADOR E DE ELETRODO DE MARCAPASSO DE CÂMARA ÚNICA	01 - Cirurgia cardiovascular	TROCA TANTO DO GERADOR COMO DO ELETRODO MALFUNCIONANTES DE MARCAPASSO DE CÂMARA ÚNICA.	R$ 271,77	R$ 644,51	R$ 916,28	2	Sim	Não	TROCA E/OU MANUTENÇÃO DE DISPOSITIVOS CARDÍACOS IMPLANTÁVEIS
04.06.01.116-8	TROCA DE GERADOR E DE ELETRODOS DE CARDIO-DESFIBRILADOR	01 - Cirurgia cardiovascular	TROCA TANTO DO GERADOR COMO DO ELETRODO MALFUNCIONANTES DE CARDIDESFIBRILADOR.	R$ 271,77	R$ 626,35	R$ 898,12	2	Sim	Não	TROCA E/OU MANUTENÇÃO DE DISPOSITIVOS CARDÍACOS IMPLANTÁVEIS
04.06.01.117-6	TROCA DE GERADOR E DE ELETRODOS DE CARDIO-DESFIBRILADOR MULTISITIO	01 - Cirurgia cardiovascular	TROCA TANTO DO GERADOR COMO DO ELETRODO MALFUNCIONANTES DE CARDIDESFIBRILADOR MULTI-SÍTIO.	R$ 271,77	R$ 626,35	R$ 898,12	2	Sim	Não	TROCA E/OU MANUTENÇÃO DE DISPOSITIVOS CARDÍACOS IMPLANTÁVEIS
04.06.01.118-4	TROCA DE GERADOR E DE ELETRODOS DE MARCAPASSO DE CÂMARA DUPLA	01 - Cirurgia cardiovascular	TROCA TANTO DO GERADOR COMO DO ELETRODO MALFUNCIONANTES DE MARCAPASSO DE CÂMARA DUPLA.	R$ 271,77	R$ 644,72	R$ 916,49	2	Sim	Não	TROCA E/OU MANUTENÇÃO DE DISPOSITIVOS CARDÍACOS IMPLANTÁVEIS
04.06.01.119-2	TROCA DE GERADOR E DE ELETRODOS NO MARCAPASSO MULTI-SITIO	01 - Cirurgia cardiovascular	TROCA TANTO DO GERADOR COMO DO ELETRODO MALFUNCIONANTES DE MARCAPASSO MULTI-SÍTIO.	R$ 271,77	R$ 626,35	R$ 898,12	2	Sim	Não	TROCA E/OU MANUTENÇÃO DE DISPOSITIVOS CARDÍACOS IMPLANTÁVEIS
04.06.01.054-4	FECHAMENTO DE COMUNICAÇÃO INTERVENTRICULAR	01 - Cirurgia cardiovascular	PROCEDIMENTO QUE CONSISTE EM CORRIGIR A COMUNICAÇÃO ENTRE OS DOIS VENTRÍCULOS, IMPEDINDO A PASSAGEM DE SANGUE DE UM VENTRÍCULO PARA O OUTRO, ATRAVÉS DO USO DE RETALHOS DE ENXERTO ORGÂNICO OU INORGÂNICO.	R$ 4.079,80	R$ 3.365,37	R$ 7.445,17	5	Sim	Sim	OUTROS PROCEDIMENTOS CARDIOVASCULARES DE CORREÇÃO
04.06.01.121-4	UNIFOCALIZAÇÃO DE RAMOS DA ARTÉRIA PULMONAR C/ CIRCULAÇÃO EXTRACORPÓREA	01 - Cirurgia cardiovascular	CONSISTE EM UNIFICAR RAMOS DA ARTÉRIA PULMONAR, CONGENITAMENTE MALFORMADOS, EM UM RAMO ÚNICO PARA MELHORAR O APORTE DE SANGUE PARA O PULMÃO. É NECESSÁRIA A CIRCULAÇÃO EXTRA-CORPÓREA NOS CASOS EM QUE A OXIGENAÇÃO DO SANGUE É MUITO BAIXA.	R$ 10.762,50	R$ 5.795,19	R$ 16.557,69	8	Sim	Sim	CORREÇÃO DE ANOMALIAS CARDÍACAS
04.06.01.122-2	UNIFOCALIZAÇÃO DE RAMOS DA ARTÉRIA PULMONAR S/ CIRCULAÇÃO EXTRACORPÓREA	01 - Cirurgia cardiovascular	CONSISTE EM UNIFICAR RAMOS DA ARTÉRIA PULMONAR, CONGENITAMENTE MALFORMADOS, EM UM RAMO ÚNICO PARA MELHORAR O APORTE DE SANGUE PARA O PULMÃO.	R$ 7.960,32	R$ 4.286,33	R$ 12.246,65	5	Sim	Sim	CORREÇÃO DE ANOMALIAS CARDÍACAS
04.06.01.123-0	ANASTOMOSE SISTEMICO PULMONAR COM CEC	01 - Cirurgia cardiovascular	PROCEDIMENTO QUE CONSISTE EM AUMENTAR O FLUXO DE SANGUE PARA OS PULMÕES, CRIANDO UM DESVIO DO SANGUE DA AORTA E SEUS RAMOS PARA AS ARTÉRIAS PULMONARES, ATRAVÉS DE CONEXÃO DIRETA NAS ARTÉRIAS PULMONARES OU COM USO DE ENXERTOS TUBULARES. FEITA COM CIRCULAÇÃO EXTRA CORPÓREA.	R$ 1.223,47	R$ 2.631,90	R$ 3.855,37	15	Sim	Sim	ANASTOMOSE
04.06.01.073-0	LIGADURA DE FÍSTULA SISTÊMICO-PULMONAR	01 - Cirurgia cardiovascular	CONSISTE NA INTERRUPÇÃO DE COMUNICAÇÕES ANORMAIS DE SANGUE ENTRE AS CIRCULAÇÕES SISTÊMICA E PULMONAR. ESTAS FISTULAS SÃO CRIADAS FISIOLOGICAMENTE ENTRE AS ARTÉRIAS E PEQUENAS VEIAS PULMONARES (NEO-VASOS) COM A FINALIDADE DE ENVIAR MAIOR QUANTIDADE DE SANGUE OXIGENADO. PRESENTE NAS PATOLOGIAS COM DIMINUIÇÃO OU AUSÊNCIA DE CIRCULAÇÃO PULMONAR (T.FALLOT, ATRESIA PULMONAR ETC...). PROMOVE-SE AS LIGADURAS DESTAS FISTULAS.	R$ 3.223,08	R$ 1.382,55	R$ 4.605,63	5	Sim	Sim	OUTROS PROCEDIMENTOS CARDIOVASCULARES DE CORREÇÃO
04.06.01.125-7	CORREÇÃO DE CORONARIA ANOMALA (19 A 110)	01 - Cirurgia cardiovascular	PROCEDIMENTO QUE CONSISTE EM REIMPLANTAR A(S) ARTÉRIA(S) CORONÁRIA(S) NA AORTA, COM OU SEM USO DE RETALHOS TUBULARES.	R$ 4.716,47	R$ 1.684,45	R$ 6.400,92	19	Sim	Sim	CORREÇÃO DE ANOMALIAS CARDÍACAS
04.06.01.126-5	ABERTURA DE ESTENOSE AORTICA VALVAR (CRIANÇA E ADOLESCENTE)	01 - Cirurgia cardiovascular	PROCEDIMENTO QUE CONSISTE EM AUMENTAR O FLUXO DE SANGUE DO VENTRÍCULO ESQUERDO PARA A AORTA, TRATANDO VÁLVULA COM FLUXO RESTRITIVO, SEM PRÓTESE VALVAR.	R$ 8.333,57	R$ 4.487,31	R$ 12.820,88	5	Sim	Sim	PROCEDIMENTOS DE CIRURGIA CARDIOVASCULAR INFANTOJUVENIL
04.06.01.127-3	ABERTURA DE ESTENOSE PULMONAR VALVAR (CRIANÇA E ADOLESCENTE)	01 - Cirurgia cardiovascular	PROCEDIMENTO QUE CONSISTE EM AUMENTAR O FLUXO DE SANGUE DO VENTRÍCULO DIREITO PARA A ARTÉRIA PULMONAR, TRATANDO VÁLVULA COM FLUXO RESTRITIVO, SEM PRÓTESE VALVAR.	R$ 7.116,60	R$ 3.832,02	R$ 10.948,62	5	Sim	Sim	PROCEDIMENTOS DE CIRURGIA CARDIOVASCULAR INFANTOJUVENIL

04.06.01.128-1	AMPLIAÇÃO DE VIA DE SAÍDA DO VENTRÍCULO DIREITO E/OU RAMOS PULMONARES (CRIANÇA E ADOLESCENTE)	01 - Cirurgia cardiovascular	PROCEDIMENTO QUE CONSISTE EM AUMENTAR O FLUXO DE SANGUE ENTRE O VENTRÍCULO DIREITO E AS ARTÉRIAS PULMONARES, ATRAVÉS DE AMPLIAÇÃO E ALARGAMENTO DE PARTE DO VENTRÍCULO DIREITO E/OU ANEL VALVAR PULMONAR E/OU ARTÉRIAS PULMONARES, COM USO DE RETALHOS DE ENXERTOS ORGÂNICOS E/OU INORGÂNICOS COM OU SEM PRÓTESES VALVARES.	R$ 10.394,49	R$ 5.597,03	R$ 15.991,52	8	Sim	Sim	PROCEDIMENTOS DE CIRURGIA CARDIOVASCULAR INFANTOJUVENIL
04.06.01.129-0	AMPLIAÇÃO DE VIA DE SAÍDA DO VENTRÍCULO ESQUERDO (CRIANÇA E ADOLESCENTE)	01 - Cirurgia cardiovascular	PROCEDIMENTO QUE CONSISTE EM AUMENTAR O FLUXO DE SANGUE ENTRE O VENTRÍCULO ESQUERDO E A AORTA, ATRAVÉS DE AMPLIAÇÃO E ALARGAMENTO DE PARTE DO VENTRÍCULO ESQUERDO E/OU ANEL VALVAR AÓRTICO, COM USO DE RETALHOS DE ENXERTOS ORGÂNICOS E/OU INORGÂNICOS, MAIS ADIÇÃO DE PRÓTESES VALVARES.	R$ 12.781,81	R$ 6.882,51	R$ 19.664,32	8	Sim	Sim	PROCEDIMENTOS DE CIRURGIA CARDIOVASCULAR INFANTOJUVENIL
04.06.01.130-3	ANASTOMOSE CAVO-PULMONAR BIDIRECIONAL (CRIANÇA E ADOLESCENTE)	01 - Cirurgia cardiovascular	PROCEDIMENTO QUE CONSISTE EM CRIAR UM ATALHO DO SANGUE ENTRE A VEIA CAVA SUPERIOR E AS ARTÉRIAS PULMONARES, ATRAVÉS DA CONECÇÃO DA VEIA CAVA SUPERIOR DIRETAMENTE NAS ARTÉRIAS PULMONARES, EM CRIANÇAS COM HIPODESENVOLVIMENTO DE UM DOS VENTRÍCULOS. ATUALMENTE PODE SER FEITA PARA DESCOMPRESSÃO DO VENTRÍCULO DIREITO EM OUTRAS DOENÇAS COMO ANOMALIA DE EBSTEIN.	R$ 9.545,53	R$ 5.139,90	R$ 14.685,43	8	Sim	Sim	PROCEDIMENTOS DE CIRURGIA CARDIOVASCULAR INFANTOJUVENIL
04.06.01.131-1	ANASTOMOSE SISTEMICO-PULMONAR (CRIANÇA E ADOLESCENTE)	01 - Cirurgia cardiovascular	PROCEDIMENTO QUE CONSISTE EM AUMENTAR O FLUXO DE SANGUE PARA OS PULMÕES, CRIANDO UM DESVIO DO SANGUE DA AORTA E SEUS RAMOS PARA AS ARTÉRIAS PULMONARES, ATRAVÉS DE CONEXÃO DIRETA NAS ARTÉRIAS PULMONARES OU COM USO DE ENXERTOS TUBULARES.	R$ 7.885,69	R$ 4.246,14	R$ 12.131,83	5	Sim	Sim	PROCEDIMENTOS DE CIRURGIA CARDIOVASCULAR INFANTOJUVENIL
04.06.01.124-9	CORRECAO DE COARCTACAO DA AORTA COM CEC	01 - Cirurgia cardiovascular	PROCEDIMENTO QUE CONSISTE EM RESSECAR OU AMPLIAR REGIÃO DA AORTA QUE ENCONTRA-SE COM OBSTRUÇÃO PARCIAL OU COM OU SEM USO DE ENXERTOS TUBULARES OU RETALHOS. FEITA COM CIRCULAÇÃO EXTRA CORPÓREA.	R$ 2.685,90	R$ 1.223,47	R$ 3.909,37	13	Sim	Sim	OUTROS PROCEDIMENTOS CARDIOVASCULARES DE CORREÇÃO
04.06.01.133-8	CORRECAO DE COARCTACAO DA AORTA (CRIANÇA E ADOLESCENTE)	01 - Cirurgia cardiovascular	PROCEDIMENTO QUE CONSISTE EM RESSECAR OU AMPLIAR REGIÃO DA AORTA QUE ENCONTRA-SE COM OBSTRUÇÃO PARCIAL, COM OU SEM USO DE ENXERTOS TUBULARES OU RETALHOS.	R$ 6.743,35	R$ 3.631,03	R$ 10.374,38	5	Sim	Sim	PROCEDIMENTOS DE CIRURGIA CARDIOVASCULAR INFANTOJUVENIL
04.06.01.134-6	CORRECAO DE DRENAGEM ANOMALA DO RETORNO SISTEMICO (CRIANÇA E ADOLESCENTE)	01 - Cirurgia cardiovascular	PROCEDIMENTO QUE CONSISTE EM REIMPLANTAR A(S) VEIA(S) CAVA(S) NO ÁTRIO DIREITO, COM OU SEM USO DE RETALHOS OU ENXERTOS TUBULARES	R$ 10.762,50	R$ 5.795,19	R$ 16.557,69	8	Sim	Sim	PROCEDIMENTOS DE CIRURGIA CARDIOVASCULAR INFANTOJUVENIL
04.06.01.135-4	CORREÇÃO DE DRENAGEM ANOMALA PARCIAL DE VEIAS PULMONARES (CRIANÇA E ADOLESCENTE)	01 - Cirurgia cardiovascular	PROCEDIMENTO QUE CONSISTE EM REIMPLANTAR A(S) VEIA(S) PULMONARE(S) NO ÁTRIO ESQUERDO, COM OU SEM USO DE RETALHOS OU ENXERTOS TUBULARES. NÃO INCLUI QUANDO TODAS AS VEIAS PULMONARES ESTÃO COM CONEXÃO ANORMAL.	R$ 8.238,57	R$ 4.436,15	R$ 12.674,72	8	Sim	Sim	PROCEDIMENTOS DE CIRURGIA CARDIOVASCULAR INFANTOJUVENIL
04.06.01.136-2	CORRECAO DE ESTENOSE MITRAL CONGENITA (CRIANÇA E ADOLESCENTE)	01 - Cirurgia cardiovascular	PROCEDIMENTO QUE CONSISTE EM AUMENTAR O FLUXO DE SANGUE DO ÁTRIO ESQUERDO PARA O VENTRÍCULO ESQUERDO, ABRINDO A VÁLVULA COM FLUXO RESTRITIVO, SEM PRÓTESE VALVAR.	R$ 12.781,81	R$ 6.882,51	R$ 19.664,32	8	Sim	Sim	PROCEDIMENTOS DE CIRURGIA CARDIOVASCULAR INFANTOJUVENIL
04.06.01.137-0	CORREÇÃO DE ESTENOSE SUPRA-AÓRTICA (CRIANÇA E ADOLESCENTE)	01 - Cirurgia cardiovascular	PROCEDIMENTO QUE CONSISTE EM AUMENTAR O FLUXO DE SANGUE DO VENTRÍCULO ESQUERDO PARA A AORTA, TRATANDO A REGIÃO ACIMA DA VALVA, QUE ENCONTRA-SE OBSTRUTIVA, PROMOVENDO AMPLIAÇÃO COM OU SEM RETALHO DE ENXERTO ORGÂNICO OU INORGÂNICO	R$ 7.116,60	R$ 3.832,02	R$ 10.948,62	5	Sim	Sim	PROCEDIMENTOS DE CIRURGIA CARDIOVASCULAR INFANTOJUVENIL
04.06.01.138-9	CORRECAO DE FISTULA AORTO-CAVITARIAS (CRIANÇA E ADOLESCENTE)	01 - Cirurgia cardiovascular	PROCEDIMENTO QUE CONSISTE EM ELIMINAR COMUNICAÇÕES ANORMAIS ENTRE OS VASOS E CÂMARAS DO CORAÇÃO	R$ 9.545,53	R$ 5.139,90	R$ 14.685,43	8	Sim	Sim	PROCEDIMENTOS DE CIRURGIA CARDIOVASCULAR INFANTOJUVENIL
04.06.01.139-7	CORREÇÃO DE HIPERTROFIA SEPTAL ASSIMETRICA (CRIANÇA E ADOLESCENTE)	01 - Cirurgia cardiovascular	PROCEDIMENTO QUE CONSISTE NA RESSECÇÃO DE UMA FAIXA MUSCULAR NA VIA DE SAÍDA DO VENTRÍCULO ESQUERDO PARA ALIVIAR A OBSTRUÇÃO CAUSADA PELO SEPTO QUE ENCONTRA-SE HIPERTROFIADO, CAUSANDO OBSTRUÇÃO SUBVALVAR AÓRTICA.	R$ 11.797,80	R$ 6.352,66	R$ 18.150,46	8	Sim	Sim	PROCEDIMENTOS DE CIRURGIA CARDIOVASCULAR INFANTOJUVENIL
04.06.01.140-0	CORRECAO DE INSUFICIENCIA DA VALVULA TRICUSPIDE (CRIANÇA E ADOLESCENTE)	01 - Cirurgia cardiovascular	PROCEDIMENTO QUE CONSISTE EM REPARAR VALVA QUE APRESENTA VAZAMENTO, SEM USO DE PRÓTESE VALVAR, MAS PODENDO USAR ANEL PROTÉTICO.	R$ 11.797,80	R$ 6.352,66	R$ 18.150,46	8	Sim	Sim	PROCEDIMENTOS DE CIRURGIA CARDIOVASCULAR INFANTOJUVENIL
04.06.01.141-9	CORRECAO DE INSUFICIENCIA MITRAL CONGENITA (CRIANÇA E ADOLESCENTE)	01 - Cirurgia cardiovascular	PROCEDIMENTO QUE CONSISTE EM REPARAR VALVA QUE APRESENTA VAZAMENTO, SEM USO DE PRÓTESE VALVAR, MAS PODENDO USAR ANEL PROTÉTICO.	R$ 10.762,40	R$ 5.795,14	R$ 16.557,54	8	Sim	Sim	PROCEDIMENTOS DE CIRURGIA CARDIOVASCULAR INFANTOJUVENIL
04.06.01.142-7	CORRECAO DE PERSISTENCIA DO CANAL ARTERIAL (CRIANÇA E ADOLESCENTE)	01 - Cirurgia cardiovascular	PROCEDIMENTO QUE CONSISTE EM INTERROMPER COMUNICAÇÃO ANORMAL ENTRE A AORTA E O TRONCO PULMONAR ATRAVÉS DE SUTURA DIRETA, GERALMENTE SEM USO DE REMENDO DE ENXERTO.	R$ 3.351,59	R$ 1.923,03	R$ 5.274,62	5	Sim	Sim	PROCEDIMENTOS DE CIRURGIA CARDIOVASCULAR INFANTOJUVENIL

04.06.01.143-5	CORRECAO DO CANAL ATRIO-VENTRICULAR PARCIAL / INTERMEDIARIO (CRIANÇA E ADOLESCENTE)	01 - Cirurgia cardiovascular	PROCEDIMENTO QUE CONSISTE EM CORRIGIR A COMUNICAÇÃO ENTRE OS DOIS VENTRÍCULOS E OS DOIS ÁTRIOS, IMPEDINDO A COMUNICAÇÃO DE SANGUE ENTRE AS CAVIDADES CARDÍACAS, ATRAVÉS DO USO DE RETALHOS DE ENXERTO ORGÂNICO OU INORGÂNICO, MAIS A CORREÇÃO DAS VALVAS QUE SÃO MAL FORMADAS, GERALMENTE SEM USO DE PRÓTESE VALVAR.	R$ 9.545,53	R$ 5.139,90	R$ 14.685,43	8	Sim	Sim	PROCEDIMENTOS DE CIRURGIA CARDIOVASCULAR INFANTOJUVENIL
04.06.01.144-3	CORRECOES DE ANOMALIAS DO ARCO AORTICO (CRIANÇA E ADOLESCENTE)	01 - Cirurgia cardiovascular	PROCEDIMENTO QUE CONSISTE EM RESSECAR OU INTERROMPER O FLUXO POR SEGMENTOS EXTRANUMERÁRIOS DE ARCO AÓRTICO OU DE VASOS DA BASE, QUE PODEM CONSTRINGIR ESTRUTURAS VIZINHAS.	R$ 8.443,77	R$ 4.546,65	R$ 12.990,42	5	Sim	Sim	PROCEDIMENTOS DE CIRURGIA CARDIOVASCULAR INFANTOJUVENIL
04.06.01.132-0	BANDAGEM DA ARTERIA PULMONAR (CRIANÇA E ADOLESCENTE)	01 - Cirurgia cardiovascular	PROCEDIMENTO QUE CONSISTE EM DIMINUIR O CALIBRE DA(S) ARTÉRIA(S) PULMONAR(ES) COM USO DE DISPOSITIVOS OU RETALHOS DE ENXERTO AO REDOR DO VASO PARA DIMINUIR O FLUXO DE SANGUE PARA O PULMÃO E/OU AUMENTAR A PRESSÃO NO VENTRÍCULO SUBPULMONAR.	R$ 7.960,32	R$ 4.286,33	R$ 12.246,65	5	Sim	Sim	PROCEDIMENTOS DE CIRURGIA CARDIOVASCULAR INFANTOJUVENIL
04.06.01.145-1	FECHAMENTO DE COMUNICACAO INTERATRIAL (CRIANÇA E ADOLESCENTE)	01 - Cirurgia cardiovascular	PROCEDIMENTO QUE CONSISTE EM CORRIGIR A COMUNICAÇÃO ENTRE OS DOIS ÁTRIOS, IMPEDINDO A COMUNICAÇÃO DE SANGUE ENTRE ELES ATRAVÉS DO USO DE RETALHOS DE ENXERTO ORGÂNICO OU INORGÂNICO	R$ 5.809,64	R$ 3.365,37	R$ 9.175,01	5	Sim	Sim	PROCEDIMENTOS DE CIRURGIA CARDIOVASCULAR INFANTOJUVENIL
04.06.01.147-8	IMPLANTE C/ TROCA DE POSICAO DE VALVAS (CIRURGIA DE ROSS) (CRIANÇA E ADOLESCENTE)	01 - Cirurgia cardiovascular	PROCEDIMENTO QUE CONSISTE EM RESSECAR A VALVA E O TRONCO PULMONAR DO PACIENTE E IMPLANTAR NO LOCAL DA AORTA, MAIS O IMPLANTE DE UM ENXERTO ORGÂNICO (HOMOENXERTO) NA POSIÇÃO PULMONAR.	R$ 7.501,80	R$ 4.321,19	R$ 11.822,99	5	Sim	Sim	PROCEDIMENTOS DE CIRURGIA CARDIOVASCULAR INFANTOJUVENIL
04.06.01.146-0	FECHAMENTO DE COMUNICACAO INTERVENTRICULAR (CRIANÇA E ADOLESCENTE)	01 - Cirurgia cardiovascular	PROCEDIMENTO QUE CONSISTE EM CORRIGIR A COMUNICAÇÃO ENTRE OS DOIS VENTRÍCULOS, IMPEDINDO A PASSAGEM DE SANGUE DE UM VENTRÍCULO PARA O OUTRO, ATRAVÉS DO USO DE RETALHOS DE ENXERTO ORGÂNICO OU INORGÂNICO.	R$ 7.116,60	R$ 3.832,02	R$ 10.948,62	5	Sim	Sim	PROCEDIMENTOS DE CIRURGIA CARDIOVASCULAR INFANTOJUVENIL
04.06.01.149-4	RESSECCAO DE MEMBRANA SUB-AORTICA (CRIANÇA E ADOLESCENTE)	01 - Cirurgia cardiovascular	CONSISTE EM RESSECAR TECIDO ANÔMALO POSICIONADO ABAIXO DA VALVA AÓRTICA E QUE PREJUDICA O FLUXO DE SANGUE.	R$ 7.116,60	R$ 3.832,02	R$ 10.948,62	5	Sim	Sim	PROCEDIMENTOS DE CIRURGIA CARDIOVASCULAR INFANTOJUVENIL
04.06.01.150-8	ANASTOMOSE SISTEMICO PULMONAR COM CEC (CRIANÇA E ADOLESCENTE)	01 - Cirurgia cardiovascular	PROCEDIMENTO QUE CONSISTE EM AUMENTAR O FLUXO DE SANGUE PARA OS PULMÕES, CRIANDO UM DESVIO DO SANGUE DA AORTA E SEUS RAMOS PARA AS ARTÉRIAS PULMONARES, ATRAVÉS DE CONEXÃO DIRETA NAS ARTÉRIAS PULMONARES OU COM USO DE ENXERTOS TUBULARES. FEITA COM CIRCULAÇÃO EXTRA CORPÓREA.	R$ 5.477,24	R$ 2.949,28	R$ 8.426,52	15	Sim	Sim	PROCEDIMENTOS DE CIRURGIA CARDIOVASCULAR INFANTOJUVENIL
04.06.01.148-6	LIGADURA DE FISTULA SISTEMICO-PULMONAR (CRIANÇA E ADOLESCENTE)	01 - Cirurgia cardiovascular	CONSISTE NA INTERRUPÇÃO DE COMUNICAÇÕES ANORMAIS DE SANGUE ENTRE AS CIRCULAÇÕES SISTÊMICA E PULMONAR. ESTAS FISTULAS SÃO CRIADAS FISIOLÓGICAMENTE ENTRE AS ARTÉRIAS E PEQUENAS VEIAS PULMONARES (NEO-VASOS) COM A FINALIDADE DE ENVIAR MAIOR QUANTIDADE DE SANGUE OXIGENADO. PRESENTE NAS PATOLOGIAS COM DIMINUIÇÃO OU AUSÊNCIA DE CIRCULAÇÃO PULMONAR (T.FALLOT, ATRESIA PULMONAR ETC...). PROMOVE-SE AS LIGADURAS DESTAS FISTULAS.	R$ 7.476,85	R$ 4.026,00	R$ 11.502,85	5	Sim	Sim	PROCEDIMENTOS DE CIRURGIA CARDIOVASCULAR INFANTOJUVENIL
04.06.01.152-4	IMPLANTE TRANSCATETER DE VÁLVULA AÓRTICA (ITVA)	01 - Cirurgia cardiovascular	CONSISTE DA INTERVENÇÃO, TRANSCATETER, COM IMPLANTE VALVAR SEM NECESSIDADE DE TORACOTOMIA E CIRCULAÇÃO EXTRACORPÓREA. INDICADO NO TRATAMENTO DA ESTENOSE AÓRTICA GRAVE EM CASO DE PACIENTE IDOSO COM CONTRAINDICAÇÃO A CIRURGIA. INCLUI PRÓTESE CARDÍACA DO TIPO BIOLÓGICA E DE APLICAÇÃO AÓRTICA, ALÉM DE CATETERES, CATETERES BALÃO, FIOS GUIA E TODOS OS MATERIAIS NECESSÁRIOS A REALIZAÇÃO DO PROCEDIMENTO.	R$ 50.461,44	R$ 6.538,56	R$ 57.000,00	5	Sim	Sim	IMPLANTE TRANSCATETER DE VÁLVULA AÓRTICA
04.06.01.151-6	CORRECAO DE COARCTACAO DA AORTA COM CEC (CRIANÇA E ADOLESCENTE)	01 - Cirurgia cardiovascular	PROCEDIMENTO QUE CONSISTE EM RESSECAR OU AMPLIAR REGIÃO DA AORTA QUE ENCONTRA-SE COM OBSTRUÇÃO PARCIAL OU COM OU SEM USO DE ENXERTOS TUBULARES OU RETALHOS. FEITA COM CIRCULAÇÃO EXTRA CORPÓREA	R$ 5.722,70	R$ 3.081,45	R$ 8.804,15	13	Sim	Sim	PROCEDIMENTOS DE CIRURGIA CARDIOVASCULAR INFANTOJUVENIL
04.06.01.070-6	INFARTECTOMIA / ANEURISMECTOMIA ASSOCIADA OU NÃO A REVASCULARIZAÇÃO MIOCÁRDICA	01 - Cirurgia cardiovascular	RESSECÇÃO DE FRAGMENTO MAL FUNCIONANTE DO MÚSCULO DO CORAÇÃO IMPLANTANDO OU NÃO PONTE/S DE ARTÉRIA/S MAMÁRIA/S OU VEIA/S SAFENA/S CASO TENHA INDICAÇÃO. REALIZADA POR TORACOTOMIA MEDIANA TRANSESTERNAL E PERICARDIOTOMIA LONGITUDINAL E COM CIRCULAÇÃO EXTRACORPÓREA, ASSOCIADA OU NÃO A SAFENECTOMIA.	R$ 2.956,37	R$ 5.371,54	R$ 8.327,91	5	Sim	Sim	OUTROS PROCEDIMENTOS CIRÚRGICOS CARDIOVASCULARES

04.06.03.015-4	FECHAMENTO PERCUTÂNEO DE COMUNICAÇÃO INTERATRIAL SEPTAL	03 - Cardiologia intervencionista	O PROCEDIMENTO CONSISTE NA CORREÇÃO DE COMUNICAÇÃO ENTRE OS DOIS ÁTRIOS, IMPEDINDO A PASSAGEM DE SANGUE ENTRE ELES, POR MEIO DO IMPLANTE PERCUTÂNEO DE DISPOSITIVO INTRACARDÍACO (OCLUSOR SEPTAL). UTILIZA-SE UM CATETER PARA A INTRODUÇÃO E LIBERAÇÃO DO OCLUSOR NO DEFEITO DO SEPTO ATRIAL, SOB ORIENTAÇÃO SIMULTÂNEA DE ECOCARDIOGRAFIA TRANSESOFÁGICA. INCLUI O OCLUSOR SEPTAL, BEM COMO OS DEMAIS PROCEDIMENTOS, MEDICAMENTOS E MATERIAIS NECESSÁRIOS. É EXCLUDENTE COM OS PROCEDIMENTOS 04.06.01.053-6 - FECHAMENTO DE COMUNICAÇÃO INTERATRIAL E 04.06.01.145-1 - FECHAMENTO DE COMUNICAÇÃO INTERATRIAL (CRIANÇA E ADOLESCENTE).	R$ 13.778,81	R$ 3.365,37	R$ 17.144,18	3	Sim	Sim	INTERVENÇÕES CARDÍACAS PERCUTÂNEAS
04.06.03.001-4	ANGIOPLASTIA CORONARIANA	03 - Cardiologia intervencionista	DILATAÇÃO DE LESÃO OBSTRUTIVA EM ARTÉRIA CORONÁRIA MEDIANTE CATETER BALÃO POR INTRODUÇÃO PERCUTÂNEA. QUANDO HOUVER CRITÉRIOS CLÍNICOS DE ELEGIBILIDADE, ESPECIFICADOS CONFORME PROTOCOLOS LOCALMENTE IMPLEMENTADOS, A ALTA DO PACIENTE PODERÁ OCORRER EM ALGUMAS HORAS APÓS O PROCEDIMENTO, DEVENDO ESTA ALTA PRECOCE OCORRER EM OBSERVÂNCIA ÀS CONDIÇÕES CLÍNICAS E À SEGURANÇA DO PACIENTE, SITUAÇÃO NA QUAL A MODALIDADE DE ATENDIMENTO DO PROCEDIMENTO SERÁ A AMBULATORIAL.	R$ 988,48	R$ 587,24	R$ 1.575,72	3	Sim	Sim	ANGIOPLASTIA
04.06.03.002-2	ANGIOPLASTIA CORONARIANA C/ IMPLANTE DE DOIS STENTS	03 - Cardiologia intervencionista	DILATAÇÃO DE LESÃO OBSTRUTIVA EM ARTÉRIA CORONÁRIA MEDIANTE CATETER BALÃO POR INTRODUÇÃO PERCUTÂNEA. QUANDO HOUVER CRITÉRIOS CLÍNICOS DE ELEGIBILIDADE, ESPECIFICADOS CONFORME PROTOCOLOS LOCALMENTE IMPLEMENTADOS, A ALTA DO PACIENTE PODERÁ OCORRER EM ALGUMAS HORAS APÓS O PROCEDIMENTO, DEVENDO ESTA ALTA PRECOCE OCORRER EM OBSERVÂNCIA ÀS CONDIÇÕES CLÍNICAS E À SEGURANÇA DO PACIENTE, SITUAÇÃO NA QUAL A MODALIDADE DE ATENDIMENTO DO PROCEDIMENTO SERÁ A AMBULATORIAL.	R$ 988,48	R$ 587,24	R$ 1.575,72	3	Sim	Sim	ANGIOPLASTIA
04.06.03.003-0	ANGIOPLASTIA CORONARIANA COM IMPLANTE DE STENT	03 - Cardiologia intervencionista	DILATAÇÃO DE LESÃO OBSTRUTIVA EM ARTÉRIA CORONÁRIA COM IMPLANTE DE STENT MEDIANTE CATETER BALÃO POR INTRODUÇÃO PERCUTÂNEA.	R$ 988,48	R$ 997,72	R$ 1.986,20	3	Sim	Sim	ANGIOPLASTIA
04.06.03.004-9	ANGIOPLASTIA CORONARIANA PRIMÁRIA	03 - Cardiologia intervencionista	DILATAÇÃO DE LESÃO OBSTRUTIVA EM ARTÉRIA CORONÁRIA NA VIGÊNCIA DE INFARTO AGUDO DO MIOCÁRDIO MEDIANTE CATETER BALÃO POR INTRODUÇÃO PERCUTÂNEA.	R$ 1.103,08	R$ 1.478,11	R$ 2.581,19	3	Sim	Sim	ANGIOPLASTIA
04.06.03.005-7	ANGIOPLASTIA COM IMPLANTE DE DUPLO STENT EM AORTA/ARTERIA PULMONAR E RAMOS	03 - Cardiologia intervencionista	DILATAÇÃO COM CATETER BALÃO DE LESÕES OBSTRUTIVAS EM AORTA, ARTÉRIA OU VEIA PULMONAR E RAMOS POR VIA PERCUTÂNEA.	R$ 988,48	R$ 587,24	R$ 1.575,72	3	Sim	Sim	ANGIOPLASTIA
04.06.03.006-5	ANGIOPLASTIA EM ENXERTO CORONARIANO	03 - Cardiologia intervencionista	DILATAÇÃO DE LESÃO OBSTRUTIVA EM PONTES DE ARTÉRIAS OU VEIAS ANASTOMOSADAS EM ARTÉRIAS CORONÁRIAS POR VIA PERCUTÂNEA.	R$ 988,48	R$ 997,72	R$ 1.986,20	3	Sim	Sim	ANGIOPLASTIA
04.06.03.007-3	ANGIOPLASTIA EM ENXERTO CORONARIANO (COM IMPLANTE DE STENT)	03 - Cardiologia intervencionista	DILATAÇÃO DE LESÃO OBSTRUTIVA EM PONTES ARTERIAIS OU VENOSAS ANASTOMOSADAS EM ARTÉRIAS CORONÁRIAS, COM IMPLANTE DE STENT, POR VIA PERCUTÂNEA.	R$ 988,48	R$ 997,72	R$ 1.986,20	3	Não	Sim	ANGIOPLASTIA
04.06.03.008-1	ATRIOSEPTOSTOMIA COM CATETER BALÃO	03 - Cardiologia intervencionista	ABERTURA OU AMPLIAÇÃO DO FORAME OVAL PATENTE POR VIA PERCUTÂNEA.	R$ 666,00	R$ 392,86	R$ 1.058,86	4	Não	Sim	INTERVENÇÕES CARDÍACAS PERCUTÂNEAS
04.06.03.009-0	FECHAMENTO PERCUTÂNEO DO CANAL ARTERIAL / FISTULAS ARTERIOVENOSAS COM LIBERAÇÃO DE COILS	03 - Cardiologia intervencionista	FECHAMENTO DO CANAL ARTERIAL POR VIA PERCUTÂNEA COM LIBERAÇÃO DE MOLA (COILS).	R$ 988,48	R$ 587,24	R$ 1.575,72	3	Sim	Sim	INTERVENÇÕES CARDÍACAS PERCUTÂNEAS
04.06.03.010-3	RETIRADA DE CORPO ESTRANHO DE SISTEMA CARDIOVASCULAR POR TÉCNICAS HEMODINÂMICAS	03 - Cardiologia intervencionista	RETIRADA DE PEDAÇOS DE CATETER OU GUIAS INTRODUZIDOS INADVERTIDAMENTE DURANTE PUNÇÃO DE VEIA PROFUNDA OU DE CATETERISMO VENOSO OU ARTERIAL, POR VIA PERCUTÂNEA.	R$ 534,52	R$ 122,20	R$ 656,72	3	Sim	Sim	INTERVENÇÕES CARDÍACAS PERCUTÂNEAS
04.06.03.011-1	VALVULOPLASTIA AÓRTICA PERCUTÂNEA	03 - Cardiologia intervencionista	DILATAÇÃO PERCUTÂNEA, COM CATETER BALÃO, DE VÁLVULA AÓRTICA ESTENÓTICA.	R$ 1.045,68	R$ 1.178,27	R$ 2.223,95	3	Não	Sim	VALVULOPLASTIA
04.06.03.012-0	VALVULOPLASTIA MITRAL PERCUTÂNEA	03 - Cardiologia intervencionista	DILATAÇÃO POR VIA PERCUTÂNEA, COM CATETER BALÃO, DE VÁLVULA MITRAL ESTENÓTICA.	R$ 1.045,68	R$ 1.178,27	R$ 2.223,95	3	Não	Sim	VALVULOPLASTIA
04.06.03.013-8	VALVULOPLASTIA PULMONAR PERCUTÂNEA	03 - Cardiologia intervencionista	DILATAÇÃO POR VIA PERCUTÂNEA, COM CATETER BALÃO, DE VÁLVULA PULMONAR ESTENÓTICA.	R$ 666,00	R$ 667,51	R$ 1.333,51	3	Sim	Sim	VALVULOPLASTIA
04.06.03.014-6	VALVULOPLASTIA TRICUSPIDE PERCUTANEA	03 - Cardiologia intervencionista	DILATAÇÃO POR VIA PERCUTÂNEA, COM CATETER BALÃO, DE VÁLVULA TRICÚSPIDE ESTENÓTICA.	R$ 1.045,68	R$ 1.178,27	R$ 2.223,95		Não	Sim	VALVULOPLASTIA
