# Supplementary material for: iCardio: Aplicação de Business Intelligence na Avaliação da Disparidade Regional da Assistência Cardiovascular com Dados do Mundo Real
Source: Arq Bras Cardiol. 2026 May 26;123(4):e20250765. [Article in Portuguese] doi: 10.36660/abc.20250765 (PMC13398835; doi:10.36660/abc.20250765)
Supplement: Material Suplementar 2 [file 0066-782x-abc-123-4-e20250765-suppl02.pdf]

Procedimento: Abertura de estenose

Região	Norte	Nordeste	Centro-Oeste	Sudeste	Sul	Brasil
Reinternação em até 30 dias do procedimento (%)	NA	14,29	NA	6,67	16,67	10,00
Reinternação em até 30 dias da alta (%)	NA	14,29	NA	13,33	16,.7	13,33
Mortalidade hospitalar (%)	NA	NA	NA	20,00	NA	10,00
Mortalidade ≤30 dias do procedimento (%)	NA	NA	NA	13,33	NA	6,67
Mortalidade ≤30 dias da alta (%)	NA	NA	NA	20,00	NA	10,00

Procedimento: Anastomose

Região	Norte	Nordeste	Centro-Oeste	Sudeste	Sul	Brasil
Reinternação em até 30 dias do procedimento (%)	9,09	NA	NA	1,45	4,76	2,29
Reinternação em até 30 dias da alta (%)	9,09	8,00	NA	11,59	14,29	10,69
Mortalidade hospitalar (%)	9,09	32,00	20,00	14,49	9,52	16,79
Mortalidade ≤30 dias do procedimento (%)	9,09	40,00	20,00	11,59	9,52	16,79
Mortalidade ≤30 dias da alta (%)	9,09	48,00	20,00	15,94	14,29	21,37

Procedimento: Angioplastia

Região	Norte	Nordeste	Centro-Oeste	Sudeste	Sul	Brasil
Reinternação em até 30 dias do procedimento (%)	7,46	5,55	13,36	7,31	10,68	8,40
Reinternação em até 30 dias da alta (%)	8,64	6,47	14,54	8,25	12,01	9,47
Mortalidade hospitalar (%)	4,25	5,02	3,63	3,42	4,26	3,96
Mortalidade ≤30 dias do procedimento (%)	4,84	5,60	4,75	4,01	4,91	4,60
Mortalidade ≤30 dias da alta (%)	5,34	6,34	5,14	4,48	5,37	5,11

Procedimento: Cardiorressecção

Região	Norte	Nordeste	Centro-Oeste	Sudeste	Sul	Brasil
Reinternação em até 30 dias do procedimento (%)	9,09	9,50	10,81	8,66	13,13	9,85
Reinternação em até 30 dias da alta (%)	9,09	14,68	13,51	13,20	18,18	14,42
Mortalidade hospitalar (%)	22,73	14,34	18,92	16,08	21,72	17,01
Mortalidade ≤30 dias do procedimento (%)	21,21	11,74	17,12	14,23	20,71	15,13
Mortalidade ≤30 dias da alta (%)	22,73	17,10	24,32	18,66	26,77	20,17

Procedimento: Cateterismo

Região	Norte	Nordeste	Centro-Oeste	Sudeste	Sul	Brasil
Reinternação em até 30 dias do procedimento (%)	NA	6,17	7,14	3,94	7,88	5,56
Reinternação em até 30 dias da alta (%)	NA	9,53	17,86	11,48	11,04	10,91
Mortalidade hospitalar (%)	NA	19,81	57,14	34,69	39,41	31,87
Mortalidade ≤30 dias do procedimento (%)	NA	17,01	39,29	28,89	37,39	27,65
Mortalidade ≤30 dias da alta (%)	NA	20,75	57,14	36,08	40,99	33,16

Procedimento: Correção de anomalias cardíacas

Região	Norte	Nordeste	Centro-Oeste	Sudeste	Sul	Brasil
Reinternação em até 30 dias do procedimento (%)	4,76	5,59	9,63	5,98	6,94	6,29
Reinternação em até 30 dias da alta (%)	6,35	9,58	12,59	10,54	11,34	10,48
Mortalidade hospitalar (%)	31,75	17,17	27,41	20,11	26,39	21,55
Mortalidade ≤30 dias do procedimento (%)	30,16	16,37	28,89	17,72	23,61	19,75
Mortalidade ≤30 dias da alta (%)	31,75	18,36	29,63	20,54	27,55	22,43

Procedimento: Implamte de DCEI

Região	Norte	Nordeste	Centro-Oeste	Sudeste	Sul	Brasil
Reinternação em até 30 dias do procedimento (%)	6,71	5,52	7,85	7,71	7,91	7,23
Reinternação em até 30 dias da alta (%)	7,50	6,51	8,97	8,91	9,09	8,36
Mortalidade hospitalar (%)	4,47	7,77	4,46	7,74	9,29	7,78
Mortalidade ≤30 dias do procedimento (%)	5,92	8,25	5,53	8,16	9,90	8,34
Mortalidade ≤30 dias da alta (%)	6,58	9,37	6,13	9,39	10,90	9,42

Procedimento: Intervenções cardíacas percutâneas

Região	Norte	Nordeste	Centro-Oeste	Sudeste	Sul	Brasil
Reinternação em até 30 dias do procedimento (%)	2,50	14,58	15,79	9,21	11,51	10,24
Reinternação em até 30 dias da alta (%)	5,00	18,75	10,53	11,32	13,67	12,46
Mortalidade hospitalar (%)	2,50	10,42	26,32	5,79	8,63	7,42
Mortalidade ≤30 dias do procedimento (%)	5,00	11,46	21,05	5,53	5,76	6,82
Mortalidade ≤30 dias da alta (%)	5,00	13,54	26,32	7,11	8,63	8,75

Procedimento: Outros procedimentos cardiovasculares de correção

Região	Norte	Nordeste	Centro-Oeste	Sudeste	Sul	Brasil
Reinternação em até 30 dias do procedimento (%)	1,54	5,16	6,60	4,66	6,17	5,19
Reinternação em até 30 dias da alta (%)	1,54	6,79	6,60	6,99	7,90	6,92
Mortalidade hospitalar (%)	4,62	6,25	5,66	8,42	9,88	7,92
Mortalidade ≤30 dias do procedimento (%)	4,62	5,43	5,66	7,53	8,40	6,99
Mortalidade ≤30 dias da alta (%)	4,62	6,52	5,66	8,78	10,62	8,32

Procedimento: Outros procedimentos cirúrgicos cardiovasculares

Região	Norte	Nordeste	Centro-Oeste	Sudeste	Sul	Brasil
Reinternação em até 30 dias do procedimento (%)	4,91	5,14	5,81	6,66	8,47	6,69
Reinternação em até 30 dias da alta (%)	7,24	8,10	9,21	10,42	12,12	10,17
Mortalidade hospitalar (%)	17,29	12,51	17,76	14,91	16,35	15,02
Mortalidade ≤30 dias do procedimento (%)	16,59	11,28	16,23	12,94	15,38	13,55
Mortalidade ≤30 dias da alta (%)	18,46	13,74	18,97	16,42	17,86	16,44

Procedimento: Plástica e/ou enxertos cardíacos

Região	Norte	Nordeste	Centro-Oeste	Sudeste	Sul	Brasil
Reinternação em até 30 dias do procedimento (%)	3,29	5,08	8,93	6,05	10,36	6,88
Reinternação em até 30 dias da alta (%)	4,53	7,98	13,69	9,30	13,69	10,11
Mortalidade hospitalar (%)	17,70	13,58	14,09	16,47	18,76	16,09
Mortalidade ≤30 dias do procedimento (%)	14,81	12,52	14,29	13,59	17,78	14,35
Mortalidade ≤30 dias da alta (%)	18,11	14,70	15,67	17,70	20,57	17,42

Procedimento: Procedimento de cirurgia cardiovascular infantojuvenil

Região	Norte	Nordeste	Centro-Oeste	Sudeste	Sul	Brasil
Reinternação em até 30 dias do procedimento (%)	3,60	5,56	8,86	5,55	6,03	5,85
Reinternação em até 30 dias da alta (%)	7,66	7,61	10,72	8,82	12,16	9,33
Mortalidade hospitalar (%)	19,82	10,31	10,96	8,45	12,16	10,38
Mortalidade ≤30 dias do procedimento (%)	18,47	9,49	10,26	6,53	9,39	8,68
Mortalidade ≤30 dias da alta (%)	19,82	11,70	11,42	9,19	13,22	11,28

Procedimento: Revasvularização miocárdica

Região	Norte	Nordeste	Centro-Oeste	Sudeste	Sul	Brasil
Reinternação em até 30 dias do procedimento (%)	4,23	6,57	7,21	6,82	9,59	7,53
Reinternação em até 30 dias da alta (%)	7,46	8,95	11,17	10,40	13,28	10,92
Mortalidade hospitalar (%)	11,07	6,16	11,88	7,51	8,70	8,02
Mortalidade ≤30 dias do procedimento (%)	9,58	5,71	11,49	6,59	8,16	7,32
Mortalidade ≤30 dias da alta (%)	12,06	6,77	13,12	8,23	9,94	8,92

Procedimento: Septectomia ventricular

Região	Norte	Nordeste	Centro-Oeste	Sudeste	Sul	Brasil
Reinternação em até 30 dias do procedimento (%)	NA	2,38	14,29	2,11	4,94	3,81
Reinternação em até 30 dias da alta (%)	NA	2,38	7,14	3,16	8,64	5,08
Mortalidade hospitalar (%)	25,00	28,57	7,14	11,58	18,52	16,95
Mortalidade ≤30 dias do procedimento (%)	25,00	21,43	7,14	7,37	16,05	13,14
Mortalidade ≤30 dias da alta (%)	25,00	28,57	14,29	12,63	18,52	17,80

Procedimento: Troca e/ou manutenção de dispositivos cardíacos implantáveis

Região	Norte	Nordeste	Centro-Oeste	Sudeste	Sul	Brasil
Reinternação em até 30 dias do procedimento (%)	3,40	4,06	3,39	3,95	6,64	4,58
Reinternação em até 30 dias da alta (%)	3,40	4,77	3,52	4,50	8,11	5,34
Mortalidade hospitalar (%)	0,97	2,35	1,08	2,13	5,07	2,79
Mortalidade ≤30 dias do procedimento (%)	0,97	2,85	1,22	2,54	5,42	3,16
Mortalidade ≤30 dias da alta (%)	1,46	3,27	1,36	3,17	5,83	3,65

Procedimento: Valvuloplastia

Região	Norte	Nordeste	Centro-Oeste	Sudeste	Sul	Brasil
Reinternação em até 30 dias do procedimento (%)	NA	6,34	16,67	4,58	7,14	5,66
Reinternação em até 30 dias da alta (%)	NA	6,34	16,67	5,06	9,74	6,47
Mortalidade hospitalar (%)	30,77	2,82	5,56	3,37	3,25	3,77
Mortalidade ≤30 dias do procedimento (%)	23,08	2,11	5,56	3,13	3,25	3,37
Mortalidade ≤30 dias da alta (%)	30,77	2,82	5,56	4,10	4,55	4,45
