# Supplementary material for: iCardio: Aplicação de Business Intelligence na Avaliação da Disparidade Regional da Assistência Cardiovascular com Dados do Mundo Real
Source: Arq Bras Cardiol. 2026 May 26;123(4):e20250765. [Article in Portuguese] doi: 10.36660/abc.20250765 (PMC13398835; doi:10.36660/abc.20250765)
Supplement: Material Suplementar 3 [file 0066-782x-abc-123-4-e20250765-suppl03.pdf]

| Procedure Code | Procedure Name | Organizational Form | Description | Hospital Service Value | Professional Service Value | Total Hospital | Average Length of Stay (days) | Allows Extended Stay | Includes Anesthesia Value | Procedure Group |
|---|---|---|---|---|---|---|---|---|---|---|
| 04.06.01.001-3 | OPENING OF INTER-ATRIAL COMMUNICATION | 01 - Cardiovascular Surgery | PROCEDURE THAT CONSISTS OF CREATING OR ENLARGING THE COMMUNICATION BETWEEN THE RIGHT AND LEFT ATRIA | R$ 7960.32 | R$ 4286.33 | R$ 12246.65 | 5 | Yes | Yes | CARDIAC RESECTION |
| 04.06.01.002-1 | OPENING OF AORTIC VALVULAR STENOSIS | 01 - Cardiovascular Surgery | PROCEDURE THAT CONSISTS OF INCREASING THE BLOOD FLOW FROM THE LEFT VENTRICLE TO THE AORTA | R$ 4079.80 | R$ 3365.37 | R$ 7445.17 | 5 | Yes | Yes | STENOSIS OPENING |
| 04.06.01.003-0 | OPENING OF PULMONARY VALVULAR STENOSIS | 01 - Cardiovascular Surgery | PROCEDURE THAT CONSISTS OF INCREASING THE BLOOD FLOW FROM THE RIGHT VENTRICLE TO THE PULMONARY ARTERY | R$ 4079.80 | R$ 3365.37 | R$ 7445.17 | 5 | Yes | Yes | STENOSIS OPENING |
| 04.06.01.004-8 | ENLARGEMENT OF RIGHT VENTRICLE OUTFLOW TRACT | 01 - Cardiovascular Surgery | PROCEDURE THAT CONSISTS OF INCREASING THE BLOOD FLOW BETWEEN THE RIGHT VENTRICLE AND THE PULMONARY ARTERIES | R$ 7357.69 | R$ 3829.47 | R$ 11187.16 | 8 | Yes | Yes | VENTRICULAR SEPTECTOMY |
| 04.06.01.005-6 | ENLARGEMENT OF LEFT VENTRICLE OUTFLOW TRACT | 01 - Cardiovascular Surgery | PROCEDURE THAT CONSISTS OF INCREASING THE BLOOD FLOW BETWEEN THE LEFT VENTRICLE AND THE AORTA | R$ 8528.04 | R$ 4321.19 | R$ 12849.23 | 8 | Yes | Yes | VENTRICULAR SEPTECTOMY |
| 04.06.01.006-4 | BIDIRECTIONAL CAVO-PULMONARY ANASTOMOSIS | 01 - Cardiovascular Surgery | PROCEDURE THAT CONSISTS OF CREATING A SHORTCUT OF BLOOD BETWEEN THE SUPERIOR VENA CAVA AND THE PULMONARY ARTERIES | R$ 6508.73 | R$ 1923.03 | R$ 8431.76 | 8 | Yes | Yes | ANASTOMOSIS |
| 04.06.01.007-2 | TOTAL CAVO-PULMONARY ANASTOMOSIS | 01 - Cardiovascular Surgery | PROCEDURE THAT CONSISTS OF CREATING A SHORTCUT OF BLOOD BETWEEN THE 2 VENA CAVAS AND THE PULMONARY ARTERIES | R$ 10762.50 | R$ 5795.19 | R$ 16557.69 | 8 | Yes | Yes | ANASTOMOSIS |
| 04.06.01.008-0 | SYSTEMIC-PULMONARY ANASTOMOSIS | 01 - Cardiovascular Surgery | PROCEDURE THAT CONSISTS OF INCREASING THE BLOOD FLOW TO THE LUNGS | R$ 3631.92 | R$ 1923.03 | R$ 5554.95 | 5 | Yes | Yes | ANASTOMOSIS |
| 04.06.01.010-2 | CARDIAC SUTURE | 01 - Cardiovascular Surgery | HEART SUTURE. | R$ 1175.18 | R$ 693.36 | R$ 1868.54 | 5 | Yes | Yes | OTHER CARDIOVASCULAR SURGICAL PROCEDURES |
| 04.06.01.011-0 | CARDIOTOMY FOR REMOVAL OF FOREIGN BODY | 01 - Cardiovascular Surgery | PROCEDURE THAT CONSISTS OF INCISING ONE OF THE HEART CHAMBERS TO REMOVE FOREIGN BODY | R$ 1175.18 | R$ 561.87 | R$ 1737.05 | 5 | Yes | Yes | OTHER CARDIOVASCULAR SURGICAL PROCEDURES |
| 04.06.01.012-9 | PLACEMENT OF INTRA-AORTIC BALLOON | 01 - Cardiovascular Surgery | PLACEMENT OF A BALLOON CATHETER FOR ASSISTANCE TO LEFT VENTRICLE FUNCTION | R$ 45.00 | R$ 0.00 | R$ 45.00 | - | No | | CATHETERIZATION |
| 04.06.01.013-7 | CORRECTION OF AORTIC ANEURYSM / DISSECTION | 01 - Cardiovascular Surgery | SURGERY CONSISTS OF CORRECTING THE ANEURYSMATIC OR DISSECTED AORTA WITH INTERPOSITION OF A TUBULAR GRAFT | R$ 2983.09 | R$ 7132.96 | R$ 10116.05 | 5 | Yes | Yes | CORRECTION OF CARDIAC ANOMALIES |
| 04.06.01.015-3 | CORRECTION OF PULMONARY ATRESIA AND INTERVENTRICULAR COMMUNICATION | 01 - Cardiovascular Surgery | PROCEDURE THAT CONSISTS OF CLOSING THE COMMUNICATION BETWEEN THE TWO VENTRICLES | R$ 14474.15 | R$ 7793.77 | R$ 22267.92 | 8 | Yes | Yes | CORRECTION OF CARDIAC ANOMALIES |
| 04.06.01.016-1 | CORRECTION OF SINGLE ATRIUM | 01 - Cardiovascular Surgery | PROCEDURE THAT CONSISTS OF CREATING A SEPTATION BETWEEN THE TWO ATRIA | R$ 9545.53 | R$ 5139.90 | R$ 14685.43 | 8 | Yes | Yes | CORRECTION OF CARDIAC ANOMALIES |
| 04.06.01.017-0 | CORRECTION OF ANOMALOUS BAND OF RIGHT VENTRICLE | 01 - Cardiovascular Surgery | PROCEDURE THAT CONSISTS OF RESECTING SEGMENTS OF RIGHT VENTRICLE MUSCLE | R$ 7116.60 | R$ 3832.02 | R$ 10948.62 | 5 | Yes | Yes | CARDIAC RESECTION |
| 04.06.01.018-8 | CORRECTION OF AORTIC COARCTATION | 01 - Cardiovascular Surgery | PROCEDURE THAT CONSISTS OF RESECTING OR ENLARGING REGION OF AORTA WITH PARTIAL OBSTRUCTION | R$ 3706.55 | R$ 1923.03 | R$ 5629.58 | 5 | Yes | Yes | CARDIAC RESECTION |
| 04.06.01.019-6 | CORRECTION OF INTERVENTRICULAR COMMUNICATION | 01 - Cardiovascular Surgery | PROCEDURE THAT CONSISTS OF CORRECTING THE COMMUNICATION BETWEEN THE TWO VENTRICLES | R$ 10220.38 | R$ 3365.37 | R$ 13585.75 | 8 | Yes | Yes | CORRECTION OF CARDIAC ANOMALIES |
| 04.06.01.020-0 | CORRECTION OF INTER-VENTRICULAR COMMUNICATION AND AORTIC INSUFFICIENCY | 01 - Cardiovascular Surgery | PROCEDURE THAT CONSISTS OF CORRECTING THE COMMUNICATION BETWEEN THE TWO VENTRICLES AND CORRECTION OF AORTIC VALVE DEFECT | R$ 6508.73 | R$ 3365.37 | R$ 9874.10 | 8 | Yes | Yes | CORRECTION OF CARDIAC ANOMALIES |
| 04.06.01.021-8 | CORRECTION OF COR TRIATRIATUM | 01 - Cardiovascular Surgery | PROCEDURE THAT CONSISTS OF RESECTING AN ANOMALOUS SEPTATION INSIDE ONE OF THE TWO ATRIA | R$ 10762.50 | R$ 5795.19 | R$ 16557.69 | 8 | Yes | Yes | CORRECTION OF CARDIAC ANOMALIES |
| 04.06.01.022-6 | CORRECTION OF ANOMALOUS CORONARY ARTERY (CHILD AND ADOLESCENT) | 01 - Cardiovascular Surgery | PROCEDURE THAT CONSISTS OF REIMPLANTING THE CORONARY ARTERY(IES) IN THE AORTA | R$ 14474.15 | R$ 7793.77 | R$ 22267.92 | 8 | Yes | Yes | PEDIATRIC AND JUVENILE CARDIOVASCULAR SURGERY PROCEDURES |
| 04.06.01.023-4 | CORRECTION OF ANOMALOUS SYSTEMIC VENOUS RETURN | 01 - Cardiovascular Surgery | PROCEDURE THAT CONSISTS OF REIMPLANTING THE VENA CAVA(S) IN THE RIGHT ATRIUM | R$ 6508.73 | R$ 3365.37 | R$ 9874.10 | 8 | Yes | Yes | CORRECTION OF CARDIAC ANOMALIES |
| 04.06.01.024-2 | CORRECTION OF PARTIAL ANOMALOUS PULMONARY VENOUS DRAINAGE | 01 - Cardiovascular Surgery | PROCEDURE THAT CONSISTS OF REIMPLANTING THE PULMONARY VEIN(S) IN THE LEFT ATRIUM | R$ 6508.73 | R$ 3365.37 | R$ 9874.10 | 8 | Yes | Yes | CORRECTION OF CARDIAC ANOMALIES |
| 04.06.01.025-0 | CORRECTION OF TOTAL ANOMALOUS PULMONARY VENOUS DRAINAGE | 01 - Cardiovascular Surgery | PROCEDURE THAT CONSISTS OF REIMPLANTING ALL PULMONARY VEINS IN THE LEFT ATRIUM | R$ 15807.24 | R$ 8511.59 | R$ 24318.83 | 8 | Yes | Yes | CORRECTION OF CARDIAC ANOMALIES |
| 04.06.01.092-7 | MYOCARDIAL REVASCULARIZATION WITH EXTRACORPOREAL CIRCULATION | 01 - Cardiovascular Surgery | IMPLANTS OF ARTERY AND/OR VEIN BRIDGES TO PERFUSE THE HEART BETTER | R$ 2956.37 | R$ 5176.36 | R$ 8132.73 | 5 | Yes | Yes | MYOCARDIAL REVASCULARIZATION |
| 04.06.01.093-5 | MYOCARDIAL REVASCULARIZATION WITH EXTRACORPOREAL CIRCULATION (2 OR MORE GRAFTS) | 01 - Cardiovascular Surgery | IMPLANTS OF MULTIPLE BRIDGES WITH EXTRACORPOREAL CIRCULATION | R$ 2956.37 | R$ 5448.80 | R$ 8405.17 | 5 | Yes | Yes | MYOCARDIAL REVASCULARIZATION |
| 04.06.01.094-3 | MYOCARDIAL REVASCULARIZATION WITHOUT EXTRACORPOREAL CIRCULATION | 01 - Cardiovascular Surgery | IMPLANTS OF ARTERY AND/OR VEIN BRIDGES WITHOUT EXTRACORPOREAL CIRCULATION | R$ 4940.43 | R$ 5176.36 | R$ 10116.79 | 5 | Yes | Yes | MYOCARDIAL REVASCULARIZATION |
| 04.06.01.056-0 | IMPLANTATION OF SINGLE-CHAMBER TRANSVENOUS CARDIOVERTER-DEFIBRILLATOR | 01 - Cardiovascular Surgery | IMPLANTATION OF SINGLE-CHAMBER ELECTRONIC DEVICE FOR TREATMENT OF TACHYARRHYTHMIAS | R$ 868.71 | R$ 1075.03 | R$ 1943.74 | 2 | Yes | No | IMPLANT OF ICD |
| 04.06.01.057-9 | IMPLANTATION OF MULTI-SITE TRANSVENOUS EPICARDIAL CARDIOVERTER-DEFIBRILLATOR | 01 - Cardiovascular Surgery | IMPLANTATION OF ELECTRONIC DEVICE FOR TREATMENT OF TACHYARRHYTHMIAS AND HEART FAILURE | R$ 1192.51 | R$ 1173.94 | R$ 2366.45 | 5 | Yes | Yes | IMPLANT OF ICD |
| 04.06.01.058-7 | IMPLANTATION OF DUAL-CHAMBER TRANSVENOUS CARDIOVERTER-DEFIBRILLATOR | 01 - Cardiovascular Surgery | IMPLANTATION OF DUAL-CHAMBER ELECTRONIC DEVICE FOR TREATMENT OF TACHYARRHYTHMIAS | R$ 854.96 | R$ 1173.94 | R$ 2028.90 | 2 | Yes | Yes | IMPLANT OF ICD |
| 04.06.01.080-3 | VALVE PLASTIC SURGERY | 01 - Cardiovascular Surgery | PROCEDURE TO RESTORE NORMAL FUNCTIONING OF CARDIAC VALVES WITHOUT USE OF PROSTHESIS | R$ 2824.37 | R$ 4409.89 | R$ 7234.26 | 5 | Yes | Yes | PLASTIC SURGERY AND/OR CARDIAC GRAFTS |
| 04.06.01.081-1 | VALVE PLASTIC SURGERY WITH MYOCARDIAL REVASCULARIZATION | 01 - Cardiovascular Surgery | CARDIAC VALVE RECONSTRUCTION SURGERY WITH SIMULTANEOUS ARTERIAL AND/OR VENOUS BRIDGES | R$ 2956.37 | R$ 6538.56 | R$ 9494.93 | 5 | Yes | Yes | PLASTIC SURGERY AND/OR CARDIAC GRAFTS |
| 04.06.03.001-5 | PERCUTANEOUS CLOSURE OF ATRIAL SEPTAL DEFECT | 03 - Interventional Cardiology | CLOSURE OF COMMUNICATION BETWEEN THE TWO ATRIA USING PERCUTANEOUS TECHNIQUE | R$ 5000.00 | R$ 2500.00 | R$ 7500.00 | 1 | No | Yes | PERCUTANEOUS CARDIAC INTERVENTIONS |
| 04.06.03.002-3 | CORONARY ANGIOPLASTY WITH STENT IMPLANTATION | 03 - Interventional Cardiology | CORONARY ANGIOPLASTY WITH STENT IMPLANTATION TO RESTORE BLOOD FLOW | R$ 3500.00 | R$ 2000.00 | R$ 5500.00 | 1 | No | Yes | ANGIOPLASTY |
| 04.06.03.003-1 | PERCUTANEOUS BALLOON AORTIC VALVULOPLASTY | 03 - Interventional Cardiology | TREATMENT OF AORTIC STENOSIS BY BALLOON DILATION | R$ 2500.00 | R$ 1500.00 | R$ 4000.00 | 1 | No | Yes | VALVULOPLASTY |
| 04.06.03.004-0 | PERCUTANEOUS BALLOON MITRAL VALVULOPLASTY | 03 - Interventional Cardiology | TREATMENT OF MITRAL STENOSIS BY BALLOON DILATION | R$ 2800.00 | R$ 1800.00 | R$ 4600.00 | 1 | No | Yes | VALVULOPLASTY |
| 04.06.03.005-8 | TRANSCATHETER AORTIC VALVE IMPLANTATION (TAVI) | 03 - Interventional Cardiology | MINIMALLY INVASIVE REPLACEMENT OF AORTIC VALVE USING TRANSCATHETER TECHNIQUE | R$ 8000.00 | R$ 3500.00 | R$ 11500.00 | 2 | Yes | Yes | PERCUTANEOUS CARDIAC INTERVENTIONS |
