# Supplementary material for: iCardio: Aplicação de Business Intelligence na Avaliação da Disparidade Regional da Assistência Cardiovascular com Dados do Mundo Real
Source: Arq Bras Cardiol. 2026 May 26;123(4):e20250765. [Article in Portuguese] doi: 10.36660/abc.20250765 (PMC13398835; doi:10.36660/abc.20250765)
Supplement: Tabela Suplementar [file 0066-782x-abc-123-4-e20250765-suppl04.pdf]

Supplementary Table 1: Regional distribution of mortality rates in cardiovascular procedures stratified by sex and age group, Brazil, 2019.

Region	Sex	Age Group	In-hospital mortality (%)	Mortality ≤30 days after procedure (%)	Mortality ≤30 days after discharge (%)
North	Males	Pediatrics	21.21	20.61	21.21
		Adults	6.40	6.15	7.33
	Females	Pediatrics	14.43	13.92	14.95
		Adults	7.18	7.69	8.49
Northeast	Males	Pediatrics	10.91	10.35	12.71
		Adults	05.03	5.23	06.03
	Females	Pediatrics	8.95	8.83	10.14
		Adults	6.62	6.62	7.70
Central-West	Males	Pediatrics	11.96	12.32	13.41
		Adults	5.41	5.77	6.54
	Females	Pediatrics	11.03	11.03	11.03
		Adults	6.94	7.41	8.33
Southeast	Males	Pediatrics	08.03	6.12	8.72
		Adults	4.76	4.94	5.78
	Females	Pediatrics	7.13	6.19	7.89
		Adults	6.20	6.11	7.28
South	Males	Pediatrics	11.31	8.54	12.06
		Adults	5.55	5.78	6.58
	Females	Pediatrics	8.86	7.63	9.67
		Adults	7.14	7.50	8.45
Brazil	Males	Pediatrics	10.22	8.66	11.20
		Adults	5.12	5.32	6.15
	Females	Pediatrics	8.58	7.86	9.38
		Adults	6.61	6.71	7.78

Source: iCardio
