# Supplementary material for: iCardio: Aplicação de Business Intelligence na Avaliação da Disparidade Regional da Assistência Cardiovascular com Dados do Mundo Real
Source: Arq Bras Cardiol. 2026 May 26;123(4):e20250765. [Article in Portuguese] doi: 10.36660/abc.20250765 (PMC13398835; doi:10.36660/abc.20250765)
Supplement: Translation

# Translation: iCardio: Applying Business Intelligence to Assess Regional Disparity in Cardiovascular Care with Real-World Data

**DOI:** 10.36660/abc.20250765i

**Authors:** Letícia Cunha Pereira de Souza, Miriam Allein Zago Marcolino, Pedro Henrique Engster, Yohan Casiraghi, Thiago Brusca da Costa Linn, Jerry Eduardo de Almeida de Bairos, Luciana Rodrigues de Lara, Nayê Balzan Schneider, Ana Paula Beck da Silva Etges, Carisi Anne Polanczyk

**Affiliations:** 1 Brasil Hospital de Clínicas de Porto Alegre (HCPA), Porto Alegre, RS – Brasil; 2 Brasil Programa de Pós-graduação em Ciências Biológicas: Bioquímica, Departamento de Bioquímica, Instituto de Ciências Básicas da Saúde, Universidade Federal do Rio Grande do Sul, Porto Alegre, RS – Brasil; 3 Brasil Instituto Nacional de Avaliação de Tecnologia em Saúde INCT/IATS, Porto Alegre, RS – Brasil; 4 Brasil Faculdade de Medicina, Universidade Federal de Ciências da Saúde de Porto Alegre, Porto Alegre, RS – Brasil; 5 Brasil Faculdade de Medicina, Universidade Federal do Rio Grande do Sul, Porto Alegre, RS – Brasil; 6 Brasil Programa de Pós-Graduação em Epidemiologia, Universidade Federal do Rio Grande do Sul, Porto Alegre, RS – Brasil; 7 Brasil Programa de Pós-Graduação em Cardiologia e Ciências Cardiovasculares, Universidade Federal do Rio Grande do Sul, Porto Alegre, RS – Brasil

**Keywords:** Cardiology, Health Care Quality Indicators, Routinely Collected Health Data

## Abstract

**Background:**

The assessment of cardiovascular care in Brazil is challenged by the country’s vast territory and the large volume of data. Business Intelligence (BI) tools may enhance the visualization and utilization of such information to support public health decision-making.

**Objectives:**

To demonstrate the practical application of the interactive dashboard iCardio in analyzing regional inequalities in high-complexity cardiovascular care in Brazil.

**Methods:**

Descriptive study based on secondary data made available by the iCardio dashboard, which integrates healthcare information from the Brazilian Unified Health System (SUS). Variables from 2019 were analyzed by region, considering service volume, mortality rates, hospital readmissions, and patient travel distance.

**Results:**

Analyses of cardiovascular surgery and interventional cardiology procedures indicated that the Southeast region exhibited the lowest mortality rates (in-hospital: 5.65%, 30 days after procedure: 5.73%, and 30 days after discharge: 6.77%). The North had the highest mortality rates (in-hospital: 7.56%, within 30 days after the procedure: 7.7%, and within 30 days after discharge: 8.7%). The South and Central-West regions registered the highest readmission rates within 30 days after the procedure (9.79% and 10.3%, respectively) and within 30 days after discharge (11.7% and 11.97%, respectively). The South also presented the highest standardized procedure rate (10.54), nearly double that of the Southeast (6.02). The Northeast showed the longest mean patient travel distance (94.59 km).

**Conclusions:**

The use of BI through the iCardio dashboard enables rapid and accessible analysis of healthcare data, with the potential to inform evidence-based decision-making, optimize resource allocation, and identify regional disparities in cardiovascular care.

## Introduction

Cardiovascular diseases (CVDs) currently represent a major burden on healthcare systems worldwide. In Brazil, CVDs stand out both in terms of mortality and financial costs, with more than R$1 billion spent annually on cardiovascular procedures by the Unified Health System (SUS).^1^ Furthermore, the cardiology literature highlights regional disparities in the provision of high-complexity services,^2^the volume of care in specialized centers,^3^ supplementary health coverage,^4^ and the distribution of healthcare professionals across the Brazilian territory.^5^Given the magnitude of these factors, improving the efficiency of health investments is of broad public interest. In this context, the use of indicator dashboards has emerged as a promising tool, simplifying access to and interpretation of information and potentially enhancing resource management and decision-making.^6-8^

Despite this potential, there is a scarcity of studies evaluating the application of dashboards with CVD-specific data in Brazil, which limits the systematic exploration of already available information to support decisions in public health. In this context, this study aims to contribute to this field by analyzing real-world data available in the interactive iCardio dashboard,^9^an initiative for organizing and visualizing data of interventional cardiology and cardiovascular surgery procedures performed in 2019 and 2020 in the Unified Health System (SUS). It was developed based on the Hospital Information System (SIH) and Mortality Information System (SIM) linked data.

iCardio provides detailed epidemiological parameters, including patient profiles, care locations, length of hospital stay, mortality, and financial resources allocated to procedures. Thus, the objective of this study was to explore the iCardio platform as a tool to assess variability in cardiovascular services across Brazilian regions.

## Methods

This is a descriptive ecological study that used the iCardio indicators dashboard as its data source. This online platform was developed by researchers from the Instituto de Avaliação de Tecnologia em Saúde (IATS), based at the Hospital de Clínicas de Porto Alegre (https://ror.org/010we4y38) as part of a project approved in a call for proposals from the National Council for Scientific and Technological Development (CNPq – grant 407282/2022-0), based on the administrative SUS’s databases. The conception, architecture, processing flows, and validation of the iCardio panel data were previously described.^10^

The panel consolidates 17 key quantitative indicators related to clinical aspects, healthcare service provision, equity, and financial sustainability, at the hospital, state, and national levels. These indicators refer to cardiovascular surgery procedures and interventional cardiology performed within the SUS, in the years 2019 and 2020, recorded on SIH and SIM linked databases. A total of 165 procedures were included (Supplementary file 1), based on the codes beginning with the organizational forms 04.06.01 (cardiovascular surgery) and 04.06.03 (interventional cardiology) from the Table of Procedures, Medications, Orthoses, Prostheses, and Special Materials of the SUS (SIGTAP). Data included all centers that performed one or more of the procedures listed above, which classifies them as High Complexity Cardiovascular Center (ACC), corresponding to units and reference centers qualified to perform procedures classified as high complexity, high technology, and high cost, according to current SUS regulations.^11^

The iCardio database allows for data stratification by gender, age group, race/skin color, region of residence, type of admission (emergency/elective), diagnosis (ICD-10), and procedure performed. Only aggregated data is presented, with no possibility of individual identification.^10^ For this study, the indicators listed in Table 1 were selected for each Brazilian region, using the locality-based analysis available on the dashboard. The age- and gender-standardized procedure rate expressed per million inhabitants was used for comparison across regions with populations exhibiting different demographic structures. As described in the descriptive material for the panel’s development,^10,12^ the direct standardization method was applied, estimating the expected procedure rates by region and state of residence of the patients, sex and age group to which they were projected based on the reference population defined by the number of inhabitants in these classes estimated for 2020 by the Brazilian Institute of Geography and Statistics.

**Table 1:** – Selected indicators of care delivery, clinical outcomes, and hospitalization costs by region of Brazil, 2019

Indicator	Measure/Unit	Purpose of Analysis
Number of patients (intervention and surgery)	Absolute number	Estimate the total treated population
Number of intervention patients	Absolute number	Characterize the specific volume of interventional procedures
Number of surgery patients	Absolute number	Characterize the specific volume of surgical procedures
Age- and sex-standardized rate per million inhabitants	Rate per million inhabitants	Compare regions independently of age structure and sex distribution
Male	Absolute number	Describe the patients’ demographic profile
Female	Absolute number	Describe the patients’ demographic profile
Main age group	Age group (%)	Identify the most affected age group
ICU admission	Percentage (%)	Assess the severity and complexity of care
Facilities	Absolute number	Estimate the available healthcare network
Inhabitants per facility	Ratio (inhabitants/unit)	Estimate service access and distribution
Mean travel distance for care	Kilometers (km)	Assess geographical accessibility
Number of patients – Different region	Absolute number	Quantify interregional displacements, considering the SUS regional division.
Patients are treated in a different region.	Percentage (%)	Proportion of patients treated outside their region of residence, according to the SUS regional division
Number of patients – Different macroregion	Absolute number	Quantify displacements between macroregions, considering the SUS regional division.
Patients treated in a different macroregion.	Percentage (%)	Proportion of patients treated outside their macroregion of residence, according to SUS regional division
Readmission within 30 days of the procedure	Percentage (%)	Evaluate the early post-procedure outcome
Readmission within 30 days of discharge	Percentage (%)	Assess the quality of care and post-discharge follow-up
In-hospital mortality	Percentage (%)	Measure lethality during hospitalization
Mortality within 30 days of the procedure	Percentage (%)	Evaluate the risk of early death after the procedure
Mortality within 30 days of discharge	Percentage (%)	Evaluate the risk of early death after hospital discharge
Mean cost per hospitalization	Brazilian reais (R$)	Estimate the average cost per treated patient
Total expenditure	Brazilian reais (R$)	Estimate aggregate financial impact

UTI: unidade de terapia intensiva. Fonte: iCardio.

Additionally, the distribution of mortality rates according to sex and age group was analyzed, with stratification between pediatric and adult patients. Furthermore, care outcomes were analyzed individually for each procedure. Only hospitalization data from 2019 were included in the analyses.

Data were extracted from the iCardio dashboard and organized into spreadsheets for descriptive analysis using Microsoft Excel (Microsoft Corporation, Redmond, WA, USA). Frequency measures (absolute values and percentages) were calculated to summarize the selected indicators. Comparisons between Brazilian geographic regions and patient profiles were conducted through comparative descriptive analysis. Since the data represent the total population of hospitalizations registered in SUS for the procedures under study, no hypothesis testing or inferential statistical methods were applied.

The study was approved by the Institutional Research Ethics Committee of The study was approved by the Institutional Research Ethics Committee of Hospital de Clínicas de Porto Alegre (CAAE 66784523.0.0000.5327). All data processing followed national data protection regulations. Only secondary, anonymized, and publicly available data were used, with no human subjects directly involved.

## Results

### Geographic distribution of cardiovascular care in Brazil

For each of the five Brazilian regions, the clinical indicators data are summarized in Table 2. When comparing the total number of patients with the number of hospitalizations recorded for interventional cardiology and cardiovascular surgery, the sum of procedures in both specialties exceeds the total number of patients treated. This discrepancy reflects the fact that some patients underwent procedures in both specialties.

**Table 2:** – Clinical and demographic profile of hospitalizations for cardiovascular procedures in the Brazilian public health system (SUS), Brazil, 2019

Indicators (2019)	North	Northeast	Central-West	Southeast	South	Brazil
**Clinical data**						
Number of patients (intervention and surgery)	4,742	30,693	10,956	71,35	43,188	160,897
Number of intervention patients	2,165	15,128	5,351	40,203	24,665	87,497
Number of surgery patients	2,653	16,119	5,825	32,647	19,893	77,128
Male	3,071	17,583	6,609	43,931	27,167	98,340
Female	1,669	13,088	4,3364	27,389	16,009	62,480
Age- and sex-standardized rate per million inhabitants	3.65	4.66	5.88	6.02	10.54	5.43
**Age Distribution of the Patients**						
< 1 year	88	451	139	921	462	2,061
1 to 8 years	181	903	306	1,741	780	3,910
9 to 17 years	82	336	85	576	246	1,324
9 to 17 years	73	370	92	529	239	1,302
27 to 35 years	114	365	160	844	423	2,175
36 to 44 years	209	1,506	448	2,608	1,460	6,229
45 to 53 years	543	3,696	1,320	8,166	4,801	18,520
54 to 62 years	1,072	6,796	2,564	17,466	10,395	38,284
63 to 71 years	1,208	7,737	2,870	19,904	12,397	44,109
72 to 80 years	813	5,485	2,037	12,875	8,398	29,606
**Racial distribution of patients**						
Yellow	105	344	97	628	400	1,574
White	408	2,484	1,559	38.412	36,553	79,406
Indigenous	7	6	9	5	16	43
Brown	3,048	14,770	5,140	21.385	2,724	47,057
Black	116	595	118	3,527	1,031	5,387
Conflict	39	65	43	436	135	707
No information	1,019	12,429	3,990	6,957	2,329	26,723

Source: iCardio. Note: The sum of patients by age group does not correspond to the total number of patients treated reported, a discrepancy explained by the specific processing adopted to adjust for inconsistencies in the birth dates recorded in the Hospital Admission Authorizations (AIHs), ensuring greater reliability in the age classification.^12^

Considering all hospitalizations nationwide, the majority of patients were white males, with a mean age ranging from 63 to 71 years. However, the predominance of white patients in the aggregate analysis largely reflects the higher concentration of care in the Southeast and South regions, where, as evidenced in the panel, there is both a greater volume of procedures and a higher proportion of the population self-identifying as white. In contrast, in the North, Northeast, and Central-West regions, there is a predominance of care provided to patients who identify as mixed-race, highlighting regional differences in the racial profile of patients served, even though no significant differences were observed regarding sex and age distribution among the regions.

In the age- and sex-stratified analysis (Supplementary Table 1), hospital mortality was consistently higher in the pediatric population compared to adults, particularly in the North region, where, among men, the rate in pediatric patients is more than double that observed in adults. Among women, this age difference is less pronounced. In contrast, when considering only the adult population, an inverse pattern is observed: in all regions of the country, women have higher mortality rates than men.

The Southeast had the highest absolute number of hospitalizations and procedures, followed by the South. However, when age- and sex-standardized rates were considered, the South exhibited the highest values. Although the Southeast and South stood out in terms of service volume, the highest proportion of intensive care unit (ICU) admissions was observed in the Northeast, as shown in Table 3, which brings together the healthcare and economic indicators.

**Table 3:** – Regional distribution of healthcare and economic indicators for hospitalizations, Brazil, 2019

Indicators (2019)	North	Northeast	Central-West	Southeast	South	Brazil
**Healthcare indicators**						
ICU admission (%)	45.79	58.21	49.49	56.04	53.39	54.69
Facilities	35	93	41	276	113	588
Inhabitants per facility	229,644	194,685	201,276	204,558	130,149	189,176
Mean travel distance for care (km)	90.77	94.59	92.23	45.86	68.91	65.77
Number of patients – Different region	1,726	14,824	3,544	24,249	14,99	59,327
Patients treated in a different region (%)	36.38	48.23	32.18	33.88	34.60	36.76
Number of patients – Different macroregion	969	7,158	3,112	10,36	4,984	26,579
Patients treated in a different macroregion (%)	20.43	23.30	28.25	14.49	11.52	16.49
Readmission within 30 days of the procedure (%)	6.05	5.64	10.30	7.08	9.79	7.75
Readmission within 30 days of discharge (%)	7.56	7.04	11.97	8.71	11.70	9.41
In-hospital mortality (%)	7.56	6.28	6.34	5.65	6.58	6.12
Mortality within 30 days of the procedure (%)	7.70	6.49	6.91	5.73	6.88	6.32
Mortality within 30 days of discharge (%)	8.70	7.52	7.72	6.77	7.81	7.32
**Mean cost per hospitalization (R$)**						
Mean cost per hospitalization (R$)	11,153.55	11,518.30	11,059.58	10,765.24	11,411.49	11,114.04
Total expenditure (R$)	54.73 M	369.54 M	133.77 M	818.89 M	538.15 M	1.92 B

ICU: intensive care unit. Source: iCardio.

In total, Brazil had 558 High-Complexity Cardiovascular (HCC) facilities, with the ratio of inhabitants per facility ranging from 130,149 to 229,644 depending on the region. The average distance traveled by patients to access care also varied geographically, with the Southeast presenting a significantly shorter mean travel distance compared with the other regions.

When stratified by region and health macro-region, the percentage of patients receiving care outside their region of residence varied by specialty. Among those undergoing cardiovascular surgery, 41.44% were treated outside their home region, and 21.16% outside their health macro-region. For interventional cardiology, these proportions were lower, with 32.46% of procedures performed outside the home region and 12.23% outside the macro-region.

The Center-West and South regions showed the highest readmission rates, both within 30 days of the procedure and within 30 days of hospital discharge. Regarding mortality, the North exhibited the highest crude rates across all three outcomes analyzed - in-hospital mortality, mortality within 30 days post-procedure, and mortality within 30 days post-discharge - as well as the longest mean hospital stay. In contrast, the Southeast presented the lowest crude mortality rates across the three outcomes.

In an analysis by procedure group (Supplementary File 2), the procedures with the highest hospital mortality rates in Brazil were catheterization (31.87%), correction of cardiac anomalies (21.55%), and cardio resection (17.01%). By region, ventricular septectomy reaches 57.14% in the Central-West, correction of cardiac anomalies reaches 31.75% in the North, and anastomosis registers 32% in the Northeast. Cardio resection presents 22.73% in the North and cardiac plastic surgery 18.76% in the South. The Northeast stands out in mortality 30 days after discharge for anastomosis (48%). However, it is important to emphasize that the analysis considered deaths occurring during hospitalizations associated with the procedures, rather than the underlying cause of death. Therefore, the results reflect in-hospital mortality associated with the hospitalization itself, and not mortality directly attributable to the procedure.

With respect to financial resources, reimbursement for the procedures performed totaled approximately R$1.9 billion during the study period. While the mean cost per hospitalization showed only modest variation across regions, regional expenditure varied widely, ranging from R$54.73 million to R$818.89 million, reflecting differences in service volume among regions.

## Discussion

Based on data from the iCardio dashboard for 2019, we conducted an analysis of regional variations in cardiovascular care, aiming to deepen the understanding of inequalities in cardiovascular assistance in Brazil; the results are summarized in the Central Illustration. Given the country’s vast territory and high heterogeneity in natural, economic, and social conditions across its regions,^13^ this type of evaluation is particularly relevant. The Unified Health System (SUS) has sought to reduce health disparities between regions and to improve coverage and access nationwide. Nevertheless, substantial variations persist in infrastructure, human resources, management capacity, and access to effective healthcare services.^14^ Thus, it is essential to consider how these regional specificities influence health indicators.

Considering the country’s overall clinical profile, comparative studies between pediatric and adult patients reinforce the data presented. The literature indicates that the mortality rate in HCC procedures and surgeries is significantly higher in children^15^ when compared to adults undergoing similar procedures. Furthermore, analysis of international databases indicates that pediatric mortality is strongly associated with the complexity of the procedure, age, and birth weight,^16^ while in adults, the risk is more influenced by acquired comorbidities and advanced age.^17^ The difference between sexes is mainly explained by different preoperative risk profiles,^18^ with mortality in women being more associated with systemic diseases and in men with direct cardiac damage. In addition, there are reports that some of the symptoms indicative of CVDs tend to be different between men and women, contributing to delays in diagnosis, less access to invasive procedures, and a worse prognosis in HCC surgeries.^19-21^ However, further investigations would be necessary to more precisely identify the causes of the differences observed in the present study.

Overall, the study showed that the North region exhibited less favorable outcomes compared to other regions. Previous analyses of cardiology indicators had already suggested a trend of poorer performance in the North, likely explained by socioeconomic factors.^1^ Moreover, this region faces limitations regarding the availability and accuracy of health system data.^22^ Similar to the North, the Northeast also tends to present critical issues in both access to and quality of medical care.^1,22^ In the present analysis, the Northeast recorded the highest proportion of ICU (intensive care unit) admissions and the greatest mean patient travel distance. Conversely, the Southeast concentrated the largest number of HCC centers, minimized patient travel, and presented the most favorable health indicators along with the lowest mean hospitalization costs. This may be explained by the efficiency of larger or high-volume hospitals, which are able to dilute fixed costs over a greater number of cases, optimize installed capacity, and achieve more efficient resource utilization.^23^

The South and the Center-West stood out for their elevated readmission rates, both within 30 days post-procedure and within 30 days post-discharge. Factors influencing this outcome include the quality of hospital discharge practices, continuity of outpatient care, and treatment adherence. Patient profiles are indeed a critical factor, as comorbidities, advanced age, and a higher prevalence of smoking also contribute to higher readmission rates, greater costs, and increased demand for hospital beds.^24^ Previous studies have shown that the South has the highest prevalence of smokers compared with other Brazilian regions,^25^ and it also has the second-oldest population in the country, with 12.1% of residents aged 65 years or older.^26^ However, these two regions diverge in terms of the proportion of patients treated outside their macro-region: the Center-West showed the highest percentage, whereas the South had the lowest. This territorial division into regions and health macro-regions, as established by SUS, is designed to optimize the planning and integration of healthcare actions and services, ensuring that resources and population needs are considered in a regionalized manner.^27,28^

The national analysis of iCardio data also revealed variations in patient travel between regions and macro-regions according to procedure type. Interventional cardiology procedures were generally performed within the patient’s own region or macro-region, suggesting a broader distribution of this type of service. In contrast, cardiovascular surgeries more frequently required patients to travel outside their region or macro-region, indicating that the locally installed capacity was often insufficient to meet the demand for more complex care.

The analysis of economic indicators revealed significant differences in total expenditure between regions, primarily related to the volume of hospitalizations, given that no relevant variations were observed in the average cost per hospitalization between them. This pattern is consistent with the SUS (Brazilian Unified Health System) financing logic, in which most hospital production is reimbursed for procedures with values previously defined in a table, which tends to equalize the average cost per hospitalization regionally, even though there are differences in the profile of cases and the complexity of care. Thus, total expenditure reflects, above all, population size^29^ and disease burden.^30^ In addition, another influencing factor is installed capacity, including the supply of beds and high-complexity services that act as referral centers and concentrate hospitalizations from other regions.^5^ The high average travel distance for care in the Northeast region may explain why, despite being the second most populous region, it is not the second with the highest total expenditure.

The real-world data analyzed in this study were made available through a Business Intelligence (BI) platform designed to facilitate data analysis and evidence-based decision-making in public health by providing more accessible and reliable information. The use of real-world evidence has gained prominence since 2007, following the International Society for Pharmacoeconomics and Outcomes Research (ISPOR) report, which encouraged its application in health technology assessment and in supporting both clinical and policy decisions.^31^ Regulatory agencies such as the U.S. Food and Drug Administration (FDA) and the Brazilian Health Regulatory Agency (ANVISA) have since endorsed the use of real-world data.^32,33^ In Brazil, DATASUS manages national information systems such as SIH and SIM, both used in this study, while SIGTAP supports logistical and managerial qualification of information.^34^

Several studies have already demonstrated the potential of BI tools in healthcare, with applications in pulmonology, cardiology, and neurology, where automated dashboards have been used to analyze therapy effectiveness, mortality rates, and economic impact.^35-38^ Notable examples include the interactive dashboard developed by Johns Hopkins University^39^ and the Brazilian Ministry of Health’s COVID-19 monitoring dashboard.^40^ These initiatives reinforce the value of BI-based platforms, not only for responding to public health emergencies but also for the continuous management of healthcare services, highlighting the importance of systematically using real-world data to generate evidence for planning and decision-making.

The SUS information systems were created for administrative purposes and are characterized by the fragmentation of information. DATASUS and other transparency initiatives provide public access to the content of each system separately, without an identifier that allows linkage between health events and overall mortality at the patient level. In this context, iCardio, using data linked across systems,^41^ is able to provide, within a single environment, pre-calculated and stratifiable clinical-epidemiological, care-related, geographic, and economic indicators.

Thus, the use of iCardio has significant potential to support clinical decision-making and public policies. Although in this study the analysis was conducted at the regional level, the panel also allows evaluations at the institutional level, enabling the monitoring of service performance, the identification of unjustified variability, and support for quality improvement initiatives. In this way, the tool can guide the organization of care networks, the definition of referral centers, and the allocation of resources, contributing to more informed, efficient, and equitable decisions in the SUS.

Although the iCardio dashboard covers data from both 2019 and 2020, only the 2019 dataset was analyzed here, since the COVID-19 pandemic profoundly altered hospitalization patterns and resource utilization, which could distort the analysis and compromise regional comparability. Finally, some limitations of this study must be acknowledged. First, the iCardio platform has not yet been integrated into official systems and is available only in Portuguese. Furthermore, the analysis relies on secondary SUS data (SIH and SIM), which are subject to underreporting and inconsistencies; in the main analysis, the data were evaluated in aggregate, without stratification by specific procedure type (e.g., angioplasty, coronary artery bypass grafting, or valve surgery), and readmission and mortality outcomes were not adjusted for procedure complexity. However, additional analyses stratified by procedure type, sex, and age group were performed and are available in the supplementary material. Finally, the exclusive use of 2019 data, while avoiding pandemic-related distortions, restricts the conclusions to the pre-COVID-19 period and presents a significant time limitation, as the platform lacks any prospect of being updated, making it impossible to analyze more recent trends.

## Conclusions

This article illustrates a practical application of automation in the evaluation of cardiovascular care delivery using real-world data, thereby advancing the digital maturity required for leveraging such information. The use of interactive dashboards, such as iCardio, enables the analysis of large datasets to become more accessible, faster, and more intuitive, thus enhancing their applicability for healthcare managers, researchers, and policymakers. This approach holds the potential to transform healthcare management by supporting more evidence-based and assertive decision-making, contributing to the identification of regional disparities, optimization of resources, and improvement of the quality of cardiovascular care nationwide. These findings reinforce the existence of inequities in the provision and utilization of high-complexity services, as previously reported in the literature, and highlight the importance of strategies aimed at promoting greater equity in access to and quality of cardiovascular care across the country.

## Supplemental Materials

Supplementary Figure 1

Supplementary Figure 2

Supplementary Figure 3

Supplementary Table
